# Functional features defining the efficacy of cholesterol-conjugated, self-deliverable, chemically modified siRNAs

**DOI:** 10.1093/nar/gky745

**Published:** 2018-08-29

**Authors:** Taisia Shmushkovich, Kathryn R Monopoli, Diana Homsy, Dmitriy Leyfer, Monica Betancur-Boissel, Anastasia Khvorova, Alexey D Wolfson

**Affiliations:** 1 Advirna, 60 Prescott Street, Worcester, MA 01605, USA; 2 Boston University, 44 Cummington Mall, Boston, MA 02215, USA; 3 University of Massachusetts Medical School, 368 Plantation Street. Worcester, MA 01655, USA

## Abstract

Progress in oligonucleotide chemistry has produced a shift in the nature of siRNA used, from formulated, minimally modified siRNAs, to unformulated, heavily modified siRNA conjugates. The introduction of extensive chemical modifications is essential for conjugate-mediated delivery. Modifications have a significant impact on siRNA efficacy through interference with recognition and processing by RNAi enzymatic machinery, severely restricting the sequence space available for siRNA design. Many algorithms available publicly can successfully predict the activity of non-modified siRNAs, but the efficiency of the algorithms for designing heavily modified siRNAs has never been systematically evaluated experimentally. Here we screened 356 cholesterol-conjugated siRNAs with extensive modifications and developed a linear regression-based algorithm that effectively predicts siRNA activity using two independent datasets. We further demonstrate that predictive determinants for modified and non-modified siRNAs differ substantially. The algorithm developed from the non-modified siRNAs dataset has no predictive power for modified siRNAs and vice versa. In the context of heavily modified siRNAs, the introduction of chemical asymmetry fully eliminates the requirement for thermodynamic bias, the major determinant for non-modified siRNA efficacy. Finally, we demonstrate that in addition to the sequence of the target site, the accessibility of the neighboring 3′ region significantly contributes to siRNA efficacy.

## INTRODUCTION

RNA interference (RNAi) is a natural mechanism for the modulation of gene expression by small interfering RNAs (siRNAs). A broad range of human diseases, including cancer, metabolic disorders, and neurodegeneration can be treated via the silencing of specific genes using siRNAs. Early attempts to harness RNAi for therapeutic development focused on lipid- or nanoparticle-formulated, minimally modified siRNAs (reviewed in (1,2)). Recently, non-formulated, conjugate-mediated delivery emerged as an alternative, clinically dominant delivery paradigm. By changing the nature of the ligand, this approach has the potential to enable targeted delivery to a variety of tissues (reviewed in (3,4)).

The functional activity of siRNAs is determined by their sequence, and a large number of powerful algorithms predicting unmodified siRNA efficacy have been developed (5–12). A variety of mathematical approaches were used for modeling siRNA efficacy. The majority of these algorithms describe datasets with a Pearson correlation coefficient of ∼0.6, and variation between the predictive power of the different models is relatively small (7). At the same time, many of these algorithms require time-consuming and multiparametric computations.

The introduction of chemical modifications into siRNAs often leads to higher efficacy of gene silencing due to enhanced siRNA cellular uptake and nuclease stability (13–15). Various degrees of 2′-sugar modifications by 2′-*O*-methyl, 2′-F and phosphorothioate substitution proved to have enhanced potency (16–19) and reduced off-target effects (19–21). Extensive siRNA chemical stabilization (22,23) is essential for conjugate-mediated *ex vivo* and *in vivo* efficacy and duration of silencing (24).

Extensive siRNA chemical modification can significantly affect silencing activity by interfering with RNAi enzymatic machinery interactions (25–27). This effect results in a decrease of the available sequence space for siRNA design and diminishes the utility of available predictive algorithms. Dar *et al*. (28) used machine learning to model the efficacy of modified siRNAs using a conglomerate dataset of all published chemically modified sequences (29). The chemically modified siRNAs included in this dataset were very heterogeneous, ranging from siRNAs bearing isolated modifications to a variety of heavily modified patterns. Furthermore, the siRNA functionality was evaluated using an array of different experimental methodologies. The diversity of this dataset limits its utility and predictive value for uniformly, heavily modified siRNAs.

Here, we synthesized a panel of 356 heavily modified, cholesterol-conjugated siRNAs capable of unassisted (gymnotic) cellular uptake (4)—self-deliverable siRNAs (sdRNAs). We evaluated sdRNA efficacy using consistent and well-controlled readouts. Using linear regression models, we identified positional base preferences and developed an algorithm that successfully described sdRNA efficacy within the training dataset. We validated the performance of the algorithm using two independent datasets of a total of ∼140 sdRNA sequences.

We further demonstrated that algorithms based on non-modified siRNAs have no predictive power for modified compounds and vice versa, indicating that the factors limiting siRNA efficacy are substantially affected by chemical modifications.

## MATERIALS AND METHODS

### sdRNA compound panel selection and synthesis

A panel of 356 sdRNAs targeting 17 genes was synthesized by TriLink (San Diego, CA, USA). Each sdRNA was designed as a duplex of a 15-nt sense strand and a 20-nt antisense strand with a 15-base pair complementary region. Antisense (guide) strand pyrimidines were 2′-fluoro modified. Sense strand pyrimidines were 2′-*O*-methyl modified. Positions 14–20 of the antisense strand and 14 and 15 of the sense strand were phosphorothioated. Positions 1, 2, 14 and 15 of the sense strand were always 2′-*O*-methyl modified. Position 1 of the antisense strand was chemically phosphorylated and fixed as 2′-*O*-methyl-U independently of the targeting sequence. Cholesterol was conjugated to the 3′ end of the sense strand through a TEG linker (Prime Synthesis, Aston, PA, USA). All sdRNAs have GC content lower than 55%. Based on an earlier analysis of a limited number of functional sdRNA, the selected sequences for the training dataset have over-representation of Us and As at certain positions. The controlled datasets used for normalization and significance analysis always incorporate similar positional bias.

### sdRNA treatment (validation datasets)

Cells were grown to 60–80% confluence and harvested by trypsinization.

sdRNA duplexes diluted in serum-free medium were mixed directly with cell suspensions prepared in growth media with 2× concentrated serum. Cells were incubated for 72 h, washed once with DPBS, and harvested in RNA lysis buffer (Ambion, 12173-011A) for further RNA purification and qPCR analysis. All passive transfections were performed in a 96-well plate format in triplicates, omitting the edge rows. Cells for transfection were generally cultured for up to 15 passages and kept in the log phase. A human adenocarcinoma HeLa cell line was used for human gene expression analysis. The effect of sdRNA on the endogenous level of mouse and rat genes was analyzed in mouse hepatoma Hepa1-6 and rat pheochromocytoma PC12 cells, respectively. HeLa and Hepa1-6 cells were transfected in EMEM media (ATCC, 30-2003), supplemented with 3% FBS (Gibco, 16140071) at 5000 cells/well. sdRNA treatment of PC-12 cells was performed alongside neuronal phenotype induction at 30 000 cells/well in RPMI media (Gibco, 11875-093), supplemented with 1% FBS and 100 ng/ml Nerve Growth Factor (NGF-7S; Sigma, N0513). PC-12 cells were grown and transfected on Collagen I-treated cell culture vessels (BD Corning, 12777-074 and 08-774-5).

### Reporter construction and efficacy data collection for the training dataset

Reporter plasmids were constructed for each gene by inserting gene fragments or 50-base target site fusions into a psiCheck-2 vector (Promega, C8021) containing independent expression cassettes for two luciferase genes—Renilla luciferase (RLuc) for monitoring mRNA change and Firefly (fLuc) for signal normalization. No repeated sequences were allowed, so overlapping sdRNA sites were trimmed and merged together, mimicking the native sequence environment. For each gene, we generated a single reporter plasmid containing all target sequences for that gene. The inserts’ length varied from 400 bp to 2 kb. The list of 50-nucleotide target regions that include the 20-nt target site and two 15-nt flanking sequences is given in Supplementary Materials (Supplementary Table S1). Each complete gene fragment was flanked at the 3′ end with a validated positive control sequence from MAP4K4 mRNA and inserted downstream of Renilla and upstream of a synthetic poly(A) site into a SgfI/NotI restriction site. The obtained reporter constructs were verified by sequencing.

For reporter transfection, HeLa cells were plated in antibiotic-free media at 2.5 × 10^6^ cells per 10 cm tissue culture dish. Each reporter was mixed with Fugene HD transfection reagent (Promega, E2311) at 2.5 μl:1 μg DNA ratio, incubated for 10 minutes, and added to the cells. After an 18-h incubation, cells were washed three times with PBS, collected by trypsinization, and mixed with diluted sdRNA compounds to obtain a final concentration of 1 μM sdRNA per 5000 cells/well of a 96-well plate. Cells were incubated for 48 h and then harvested in 60 μl Glo lysis buffer (Promega, E266A) added directly to each well. Renilla and Firefly luciferase assays were performed on two separate 20 μl lysate replicas from the same samples. Renilla assay buffer (Matthews buffer (30) with freshly added h-Coelenterazine) was mixed with the cell lysate at 3:1 ratio, and light emission was collected after a 3-min incubation. Firefly assay buffer (25 mM glycylglycine, 15 mM MgSO_4_, 4 mM EGTA, 1 mM DTT, 2 mM ATP, 15 mM K_2_PO_4_, pH 7.8 and 1 mM D-luciferin) was added to the duplicate lysate aliquots at the same ratio and incubated for 10 min prior to luminescence measurement. D-Luciferin was obtained from Promega (E1605), and h-Coelenterazine was obtained from NanoLight (301). Luminescence was measured on a SpectraMax i3 (Molecular Devices) with 90% gain and 0.1 sec integration time and was normalized and expressed as a percentage of untreated control. The optimized screening assays were performed with high accuracy and reproducibility with the following parameters: the average screening CV% = 6.3%, S/B = 4.3, and *Z*′ factor = 0.69. *Z*′ factor was calculated (Equation 1), where σ_+_ and σ_–_ represent the standard deviation of the positive and negative control. μ_+_ and μ_–_ represent the average of the positive and negative controls, respectively.

(1) \begin{equation*}Z{\rm{^\prime\ }} = {\rm{\ }}1 - {\rm{\ }}\frac{{3\left( {{\sigma _ - } + {\sigma _ + }} \right)}}{{\left| {{\mu _ + } - {\mu _ - }} \right|}}\end{equation*}

### qPCR assay

Total RNA was purified from transfected cells using a PureLinkTM Pro96 kit (Ambion, 12173-011A) according to the manufacturer's instructions. 30 ng total RNA was mixed with Quanta qScript XLT ToughMix (VWR, 89236672) and with fluorescent FAM-labeled specific assay and VIC-labeled probe for the reference (housekeeping) gene. RNA was then subjected to reverse transcription and qPCR in a one-step multiplex reaction using the StepOnePlus Real-Time PCR instrument (Applied Biosystems, Foster City, CA, USA), with the cycling parameters recommended for the XLT ToughMix. The following Taqman gene expression assays were used: human STAT3-FAM (Hs00374280_m1), mouse Smo-FAM (Mm01162710_m1), rat Tsc1-FAM (Rn00573107_m1), Klf4-FAM (Rn00821506_g1), and Klf9-FAM (Rn00589498_m1). Assays for reference genes were human PPIB-VIC (Hs00168719_m1), mouse Gapdh-VIC (Applied Biosystems, 4352339E), and rat beta-Actin (Rn00667869_m1). A standard curve was generated for each gene on every assayed plate by including 5-fold dilutions of RNA from untreated samples. The curves showed amplification efficiency 100 ± 10% with *R*^2^ > 0.99. Gene expression data was normalized to the appropriate internal reference, adjusted according to the standard curve, and logged as a percentage of untreated control.

### Generating the weight matrix

The weight matrix is a per-position base frequency matrix representing the linear regression algorithm that was used to score sequences for sdRNA functionality prediction. The matrix recapitulates base preferences in the 50-base region (20-base siRNA-targeting site, surrounded by 15-base flanking regions). The weight matrix was generated using functionality data from the training set of 50-base sequences and their corresponding sdRNAs. Cutoffs were selected to bin functional and non-functional 50-base sequences. Several functional cutoffs were considered, with sequences inducing more than 83% silencing (less than 17% gene expression remaining), 76% silencing (<24% gene expression remaining), and 65% silencing (<35% gene expression remaining). Non-functional sequences were defined as compounds inducing <56% silencing (>44% of gene expression remaining). Per-position base frequencies were computed for each cutoff as well as for the total training dataset.

To determine which of the 50 positions were important to predicting sdRNA functionality, corresponding test statistics were computed. Two sets of random sequences corresponding to the functional (F) and non-functional (NF) cutoffs were generated using a pseudo-random number generator (NumPy Version 1.14.2). Both datasets were generated with the same per-position composition as that of the entire dataset, with total sequences generated equal to the number of sequences in the total dataset (*N* = 356) multiplied by the cutoff sizes for functional (*q*, ranges between 41 and 138) or non-functional (*z* = 157), respectively. The standard deviations (σ) of the computed frequencies for each base at each position were computed for each randomly generated dataset. Test statistics were generated comparing the computed standard deviations to the corresponding per-position base frequency medians (*M*_d_) of the functional or non-functional training datasets (Equation 2). A one-sample *t*-test was conducted for each base at each position to compute a *P*-value from the test statistic, testing the hypothesis that selecting for (or against) a particular base at a particular position in a sequence increases the likelihood that the sequence is active (one-sample *t*-test (R Version 3.4.1) with Bonferroni correction).

A per-position, nucleotide-base matrix was then generated. For bases and positions with corresponding *P*-values that were found to be significant (*P* < 0.001 after Bonferroni correction), the weight values for their corresponding positions in the weight matrix were calculated by subtracting the per position base frequency of non-functional sequences from that of functional sequences. Positions above the cutoff (*P* > 0.001) were set to zero.

For the control, average RefSeq database (31) frequencies adjusted for initial bias in the training set were used instead of per position base frequencies from the training dataset. All other computations were performed as above to generate the control weight matrix.

(2) \begin{equation*}test{\rm{\ }}statistic\ = {\rm{\ }}\frac{{\frac{{M{d_{NF}}}}{z} - {\rm{\ }}\frac{{M{d_F}}}{q}}}{{\sqrt {\frac{{\left( {\frac{{\sigma _{NF}^2}}{{{z^2}}}} \right)}}{z} + \frac{{\left( {\frac{{\sigma _F^2}}{{{q^2}}}} \right)}}{q}} }}\end{equation*}

### Algorithm development

Linear regression-based scoring was used to derive the prediction algorithm. Final scores for each sequence were computed as a sum of the scores for every position. For the control, an algorithm was derived using a control positional preference matrix developed as described above. To avoid including artefacts from the cloning of multiple distant target sites, the positional preference matrix including 48 bases (excluding positions 1 and 50) was used for primary algorithm development for the qPCR validation dataset. In Figure 4, prediction algorithms for sdRNA and siRNA were generated using a positional base-preference matrix for the targeting (20 nucleotide) region only.

### Computing AU positional preferences

AU preferences in functional versus non-functional sequences were calculated as a sum of A and U preference in the same position and/or four-nucleotide sliding window. This value was used as a simple proxy for thermodynamic stability (32).

To calculate the significance of the observed preference in AU distribution, the random AU distribution background was calculated. Sets of random sequences each equal in size (*N* = 356) and per position base frequency to that of the training set were generated. This process was repeated to generate N random sequence sets, resulting in N*N total random sequences generated. For each set of *N* random sequences, sequences were randomly selected to generate two subsets corresponding (and equal in size) to the functional (91 sequences) and non-functional (157 sequences) datasets. The per position and/or four-nucleotide sliding window AU preferences for each set were computed by comparing the two subsets in the same way non-functional and functional sequences were compared above. The random AU background was computed by compiling the AU preferences from each set and computing the 80% confidence intervals at each position.

## RESULTS

### Evaluating the efficacy of a panel of 356 chemically modified, self-delivering siRNAs

**Figure 1.**
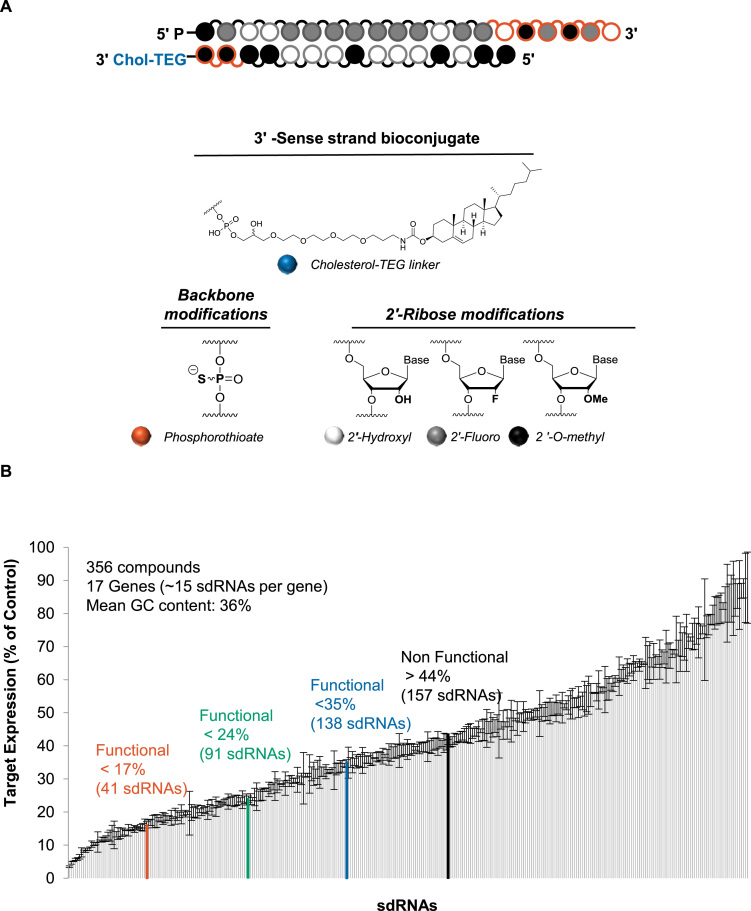
Efficacy distribution of the panel of chemically modified, asymmetric, self-delivering siRNAs (sdRNAs). (**A**) sdRNAs are asymmetric siRNAs, consisting of a 20-nucleotide antisense strand and a 15-nucleotide sense strand, in which all pyrimidines are 2′-fluoro (antisense) and 2′-*O*-methyl (sense) modified. The 3′ terminal backbone is phosphorothioated (six linkages in antisense and two in sense). The 3′ end of the sense strand is conjugated to cholesterol. (**B**) The efficacy of 356 sdRNAs targeting 17 genes was evaluated using dual luciferase reporter in HeLa cells at 1 μM (passive uptake) at 48 h (*n* = 3, mean ± SD).

sdRNAs are substantially chemically modified, cholesterol-conjugated, asymmetric siRNAs, capable of unassisted cellular uptake (33–35). sdRNAs are comprised of a 20-base antisense strand duplexed with a shorter 15-base sense strand (Figure 1A). The six 3′ nucleotides of the antisense strand and two 3′ nucleotides of the sense strand are phosphorothioated. All pyrimidines are modified with 2′-fluoro and 2′-*O*-methyl in the antisense and sense strands, respectively. In addition, some purines in both strands are 2′-*O*-methyl modified to eliminate the presence of unmodified ribose stretches. The 5′ end of the antisense strand is chemically monophosphorylated and is fixed as 2′-*O*-methyl uridine (U). In addition, positions 1 and 2 of the sense strand are always 2′-*O*-methyl modified (20), which, in combination with the shorter sense strand length, creates chemical asymmetry and prevents the sense strand from loading into the RISC complex. When conjugated to cholesterol, these compounds efficiently enter all cell types without requiring a delivery vehicle by a subset of the endocytosis mechanism associated with EEA1 (35). The general chemical configuration of the sdRNA is shown in Figure 1A.

The training dataset used for algorithm development consists of 356 sdRNA target regions across 17 different genes (∼20 sdRNAs per gene). For functional evaluation, for each target gene the sdRNA target regions of 50 bases (including 20-base siRNA targeting sites and 15-base flanking regions) were fused and cloned into the 3′ UTR of the psiCHECK-2 vector (see Methods). All 17 reporters contained an embedded universal positive control sequence for cross-assay data comparison and validation. The corresponding target sequence and efficacy of all tested compounds are shown in Supplementary Table S1.

The sdRNA dataset was designed with additional restrictions on the sequence space, including low GC content (<55%, Supplementary Figure S1A), restriction of sequences containing stretches of four or more cytosines and guanines and five or more uridines and adenines, etc. In addition, sequences with potential cross-reactivity to other genes (perfect homology to positions 2–17 of the antisense strand) and containing miRNA seeds (miRBase (36)) were excluded.

Figure 1B shows the efficacy distribution for the 356 sdRNA dataset. Compound efficacies were normalized to corresponding non-targeting controls. Although the design was originally biased toward low GC content, a well-established factor favoring siRNA efficacy (37), the fraction of highly active sequences appeared to be significantly lower than that in the context of non-modified siRNAs (5,37). Only 3% of tested sequences induced more than 90% silencing. In the published randomly-selected non-modified siRNA dataset from Huesken *et al.* (5), as many as 16% of tested compounds demonstrated similar activity (Supplementary Figure S1C). Although direct quantitative comparison of these datasets is not possible due to the differences in experimental conditions, this result is consistent with known observations that extensive chemical modification is not well-tolerated by many siRNA sequences (27,38,23), emphasizing the need for the development of a proper prediction procedure.

For algorithm development, the sdRNA dataset was subdivided into non-functional (157 sdRNAs, >44% target gene expression remaining) and functional subsets. Three functional cutoffs were used with increasing stringency: <35% (138 sdRNAs), <24% (91 sdRNAs) and <17% (41 sdRNAs) target gene expression remaining (Figure 1B). The selection of multiple functionality cutoffs allows for the identification of an optimal balance between increasing the training dataset size and minimizing the false positive rate.

### A linear regression-based algorithm for chemically modified asymmetric siRNAs

Non-modified siRNA efficacy is defined by the siRNA sequence itself. Many different mathematical models have been used to describe the relationship between siRNA sequence and efficacy, with nucleotide positional frequency being the essential parameter in all (see Introduction). Here we used per-position base preferences and linear regression to generate an siRNA prediction algorithm. This approach provided similar predictive power to other methodologies (5–7) and enabled clear visualization of the key parameters contributing to the selection process.

**Figure 2.**
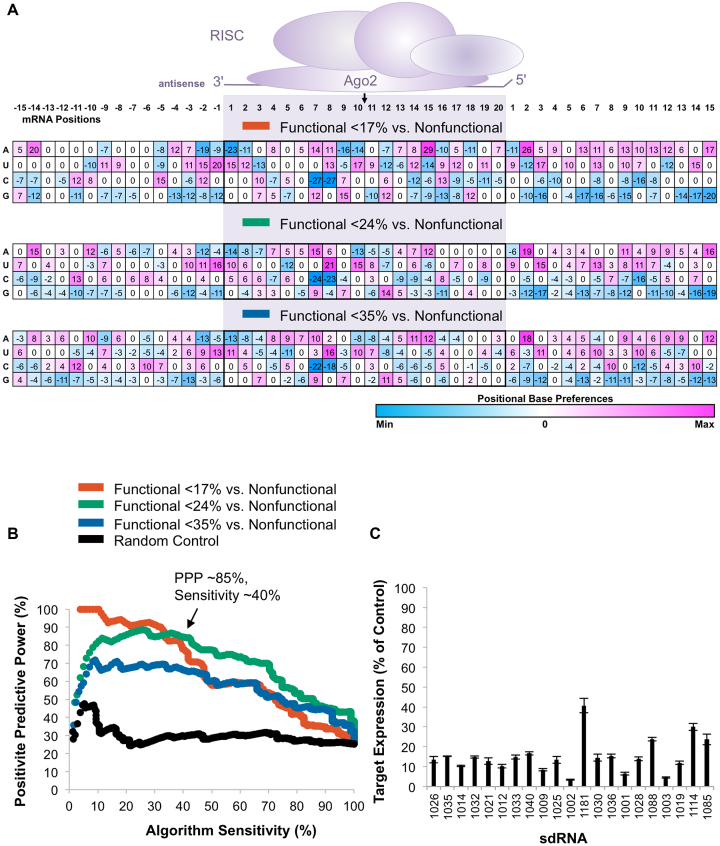
Development of an algorithm for the prediction of sdRNA efficacy. (**A**) The positional base preference matrix was generated using three functionality cutoffs (17%, 24% and 35% functional versus >44% non-functional compounds) for the 50-base regions comprising the siRNA-targeting site. Matrix weight values are color-coded by value as indicated by color bar below matrices. Analyzed mRNA positions corresponding to siRNA-targeting region (shaded) are indicated at the top. The location of cleavage site between positions 10 and 11 is indicated with a black arrow. (**B**) Using linear regression analysis (R 3.4.1), the scoring algorithm was generated for shown positional preference matrices (*P* < 0.001, see Materials and Methods). Algorithm performance is visualized as positive predictive power (PPP) versus sensitivity curves. PPP is calculated as the percent of correctly predicted (functional) sequences versus total predicted sequences for each algorithm value. Sensitivity is calculated as the percent of functional sequences selected vs total functional sequences present in the dataset for each algorithm value. sdRNA compounds with >44% gene expression remaining were defined as non-functional. The 17%/NF-preference matrix-based algorithm demonstrates the best performance with 96% PPP at 25% sensitivity. Black line shows performance of the control algorithm (see Methods). (**C**) The efficacy of individual sdRNA compounds selected by the 17%/NF scoring algorithm at 25% sensitivity (*n* = 3, mean ± SD). sdRNA IDs are indicated along the x-axis.

Figure 2A shows a positional base preference matrix computed using three functional cutoffs of different stringencies. Weights for each base and position were computed by comparing the per-position base frequencies of the functional and non-functional sdRNA subsets (see Materials and Methods). The significance of the weight parameter with respect to siRNA functionality for each base was calculated using a one-sample *t*-test (see Materials and Methods), and non-significant values were substituted with zero. Positive weights indicate preferential occurrence of a base at a particular position in functional sdRNAs, while negative numbers indicate preferential occurrence of a base at a particular position in non-functional sdRNAs. Non-zero weights are indicated in the matrix table and color-coded to reflect their magnitude. The positional preferences appear to be mostly consistent for all three efficacy cutoffs used. The most prominent features of the matrix (with the highest or lowest weights) were observed at positions 7–15, a region that also encompasses the cleavage site (between positions 10 and 11 of the 20 base siRNA targeting region (39)). For analysis, we included additional sequences immediately adjacent to the targeting region aiming to detect their potential contribution or use as an embedded internal control. Although it is generally believed that the siRNA sequence itself is a primary determinant of siRNA efficacy, we observed several highly statistically significant base preferences outside the RISC-interacting region.

A linear regression model was generated using an algorithm that incorporates the per-position base preferences from the training dataset (see Methods). Algorithm performance on a dataset was assessed by comparing positive predictive power (PPP) to sensitivity (Figure 2B). PPP is calculated as a percent of correctly predicted (functional) sequences vs total predicted sequences for each computed score. Sensitivity is calculated as a percent of functional sequences selected versus total functional sequences present in the dataset for each computed score. For comparison, the results are also displayed as Receiver Operating Characteristic (ROC) curves and presented in Supplementary Figure S2.

The <24% cutoff-based matrix was selected for further evaluation because it shows ∼85% accuracy with 40% sensitivity. The Pearson correlation between the algorithm score value derived using the selected matrix and target gene expression was 0.55 on the training dataset. As a control, we generated regression models based on an equal number of randomly selected sequences distributed in similar sized groups. The control showed no predictive power (see Materials and Methods). Thus, the linear regression of per-position base preferences adequately identifies active and inactive sdRNA sequences. Figure 2C shows the efficacy of sdRNAs predicted to be functional based on the linear regression model.

### Validation of modified siRNA algorithm through performance on independent datasets

**Figure 3.**
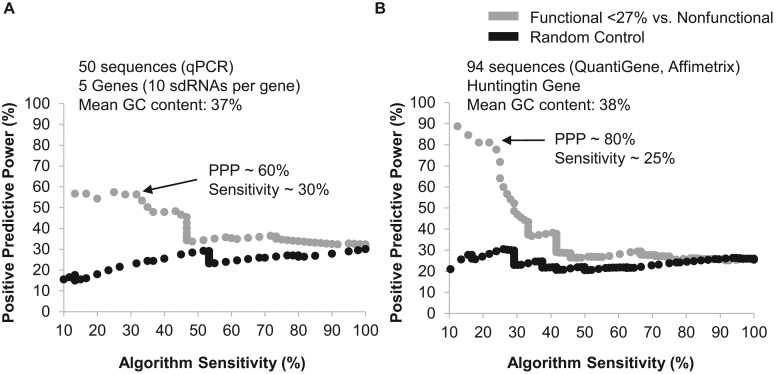
sdRNA algorithm accurately predicts efficacy using two independent datasets. The sdRNA (24%/NF) algorithm was applied to predict the efficacy of (**A**) 50 sdRNAs targeting five genes (qPCR, sdRNAs inducing ∼ <25% target gene expression are defined as functional). sdRNA algorithm predicts efficacy with ∼60% accuracy at ∼30% sensitivity. (**B**) 94 sdRNAs targeting the huntingtin gene (QuantiGene (34), sdRNAs inducing ∼ <25% target gene expression are defined as functional). sdRNA algorithm predicts efficacy with ∼80% accuracy at ∼25% sensitivity. Black line shows performance of the control algorithm (see Methods).

To validate the developed algorithm, we used two independent datasets generated using the same chemical scaffold as described in Figure 1A. The first dataset comprises 50 sequences targeting five genes (10 sdRNAs per gene), for which efficacy was measured using qPCR in several cell lines (see Materials and Methods). The second dataset was previously published and includes 94 sdRNAs targeting Huntingtin, for which sdRNA efficacy was measured using a QuantiGene Assay (34). Our algorithm effectively predicted sdRNA activity with approximately 60% predictive power at 25% sensitivity (Figure 3A and B). Construction of the validation dataset was fully independent from that of the training dataset. sdRNA efficacy was measured using direct measurement of endogenous mRNA with two technical platforms for six different genes. The predictive power was lower than shown with the training set (60% versus 80%), which is expected and in line with the predictive power and performance of published siRNA algorithms (5–7). This outcome confirms that a linear regression-based algorithm allows the effective scoring of sdRNAs with more than half of predicted compounds being functional.

### Non-modified siRNA-based algorithm has no predictive power for heavily modified siRNAs

**Figure 4.**
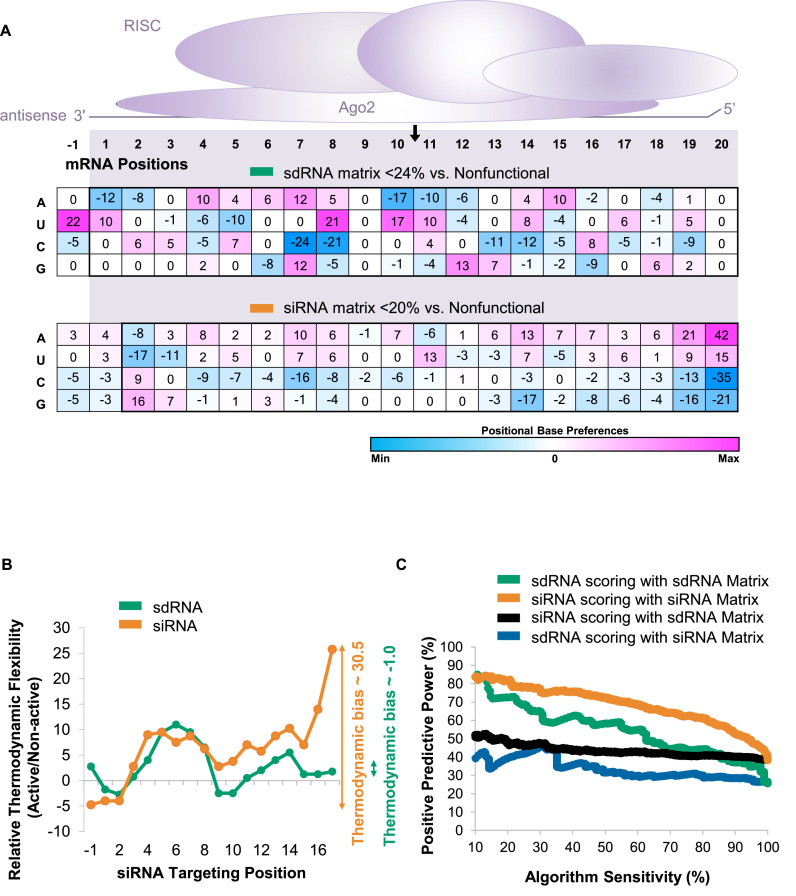
Algorithms derived from naked siRNA do not have predictive power for modified (sdRNAs) and vice versa. (**A**) The positional base preference matrix was generated from non-modified (orange) (5) and chemically modified (green) sdRNA. Sequences were aligned based on the 5′ end of the antisense strand. Matrix weight values are color-coded by value as indicated by shaded bar below the matrices. Analyzed mRNA positions corresponding to siRNA-targeting region (shaded) are indicated at the top. Black arrow indicates the location of cleavage site between positions 10 and 11. (**B**) The ability of algorithms derived from non-modified and modified sdRNA datasets to predict the efficacy of non-modified and modified siRNAs was calculated using PPP vs sensitivity plots. (**C**) The thermodynamic flexibility of the non-modified and chemically modified siRNAs was estimated by averaging GC content over a sliding window of four bases. Thermodynamic bias is indicated as the difference between the relative thermodynamic flexibility at 5′ and 3′ ends of the siRNA duplex. Chemically modified siRNAs do not display conventional thermodynamic bias.

One of the major determinants of unmodified siRNA efficacy is the thermodynamic bias defining the nature of the strand entering the RISC (32,38). The asymmetric nature of sdRNA, in combination with chemical modifications, effectively precludes the sense strand from RISC entry and, theoretically, should eliminate the effect of this parameter. Thus, position-based algorithms developed for the prediction of non-modified siRNA efficacy might not be suitable for prediction with heavily modified sdRNAs. To test this hypothesis, we generated a positional scoring matrix using the same methodology for a dataset of 2384 siRNAs from Huesken *et al*. (5) (Supplementary Figure S1B and C) and compared it to the sdRNA positional matrix. For this comparison, the analysis was restricted to the 20-base targeting region alone, as no flanking regions were included in the reporter construct in the Huesken *et al*. dataset. Figure 4A shows that base-preference matrices for non-modified and modified siRNAs differ substantially. As expected, the most prominent positional base preferences observed in the non-modified siRNA dataset are related to the introduction of a thermodynamic bias, with a strong preference toward A and U at the positions corresponding to the 5′ end of the antisense strand. These features were completely lacking in the sdRNA matrix (Figure 4B). At the same time, certain nucleotide preferences observed around the cleavage site (positions 7, 8 and 11) were similar between the datasets, possibly reflecting the general nucleotide preferences imposed by the RISC complex and potentially related to dissociation of the product upon cleavage (38,40). No other significant resemblances were observed.

Considering these differences, it is not surprising that the linear regression-based algorithm derived from non-modified siRNAs adequately described itself but failed to predict the efficacy of the modified siRNAs dataset and vice versa (Figure 4C). Consequently, unmodified siRNA selection algorithms had no predictive power for the selection of heavily chemically modified siRNA compounds.

### Regions neighboring the siRNA-targeting site contribute to efficacy

**Figure 5.**
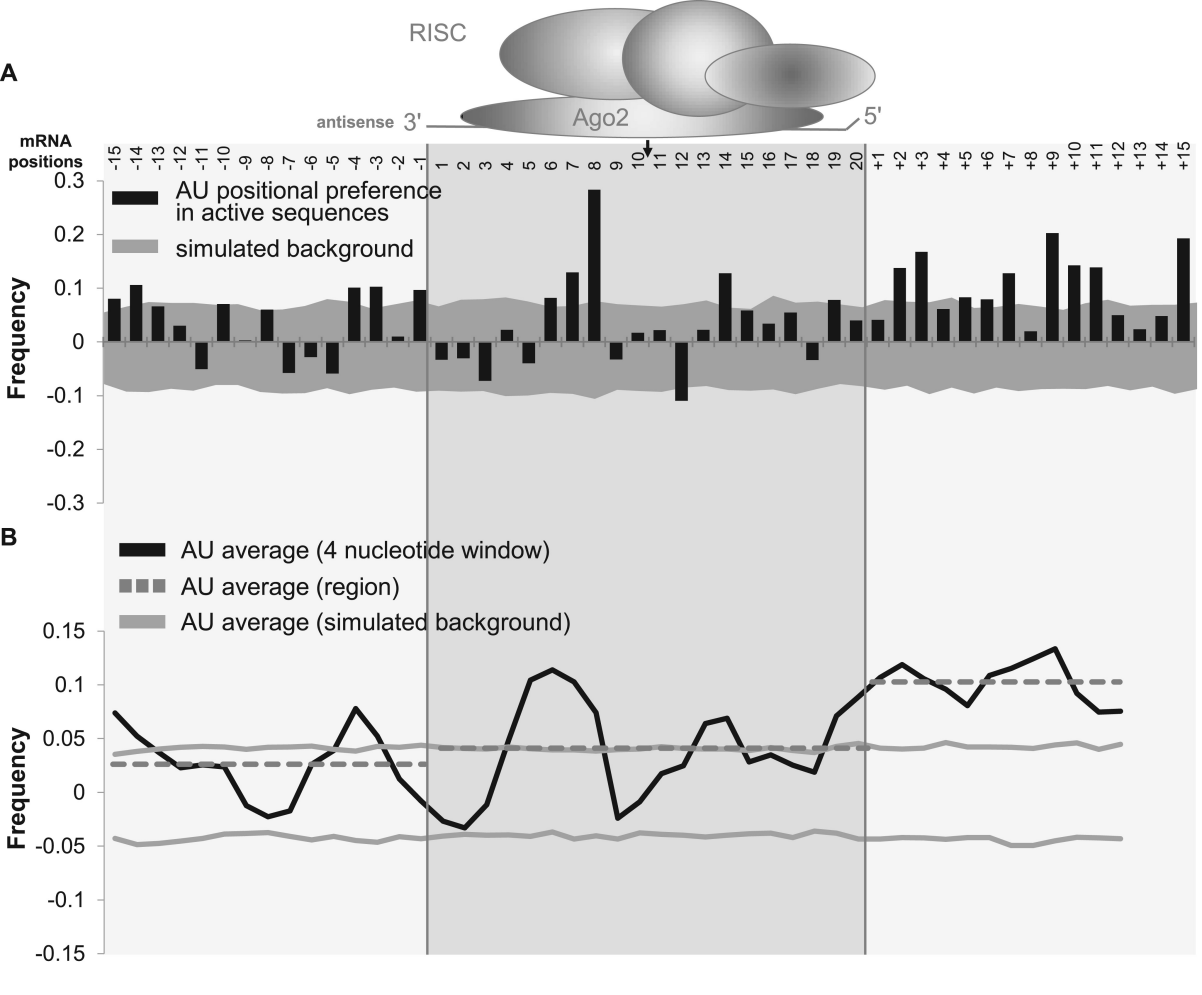
mRNA local thermodynamic flexibility in the 3′ region outside the siRNA-targeting site contributes to sdRNA efficacy. (**A**) The frequency of AU at each position (black bars) in the siRNA-targeting region and surrounding 5′ and 3′ regions was computed by subtracting the frequency of AU in non-functional siRNAs from that in functional (< 24% mRNA expression remaining) siRNAs. The background (grey area) was simulated using AU frequency in the randomly distributed training dataset of 356 siRNAs. The 80% confidence interval of the simulated background is shown. Analyzed mRNA positions are indicated at the top along with corresponding siRNA-targeting region (shaded area; positions 1-20). The location of cleavage site between positions 10 and 11 is indicated with a black arrow. (**B**) The frequency of AU at each position was averaged over a four-base region (black line). The average AU frequency was computed over each region (grey dotted line). The background (grey solid line) was averaged over a four-base region.

The positional base preference matrix (Figure 2A) contained several strong determinants located outside of the 20-base targeting region. Previously, the mRNA secondary structure around the siRNA targeting site has been proposed as important for siRNA activity (41–43). The propensity of RNA to form secondary structures is mostly defined by local GC content. Figure 5A shows calculated AU preferences for the sdRNA dataset, including regions flanking the siRNA-targeting region. The level of background noise is visualized by grey areas, corresponding to the 80% confidence interval derived from AU background simulation (see Methods). Individually, there are several positions displaying strong AU preference in the RISC-targeting region at positions 6, 7, 8 and 14. In addition, several positions outside the RISC-binding site, specifically on the 3′ end, display a preference for AU bases. Figure 5B shows an analysis of the local thermodynamic flexibility of the siRNA-targeting region along with the flanking regions. It is clear that high AU content 3′ to the targeting site is one of the most significant contributors to sdRNA functional activity, since AU preference in this region is more pronounced than in the siRNA-targeting region itself. The thermodynamic flexibility (measured as AU preference (32)) 5′ of the targeting site reaches statistical significance above the background but is less distinct.

### Position 14 of the antisense strand does not tolerate 2′-O-methyl modification

**Figure 6.**
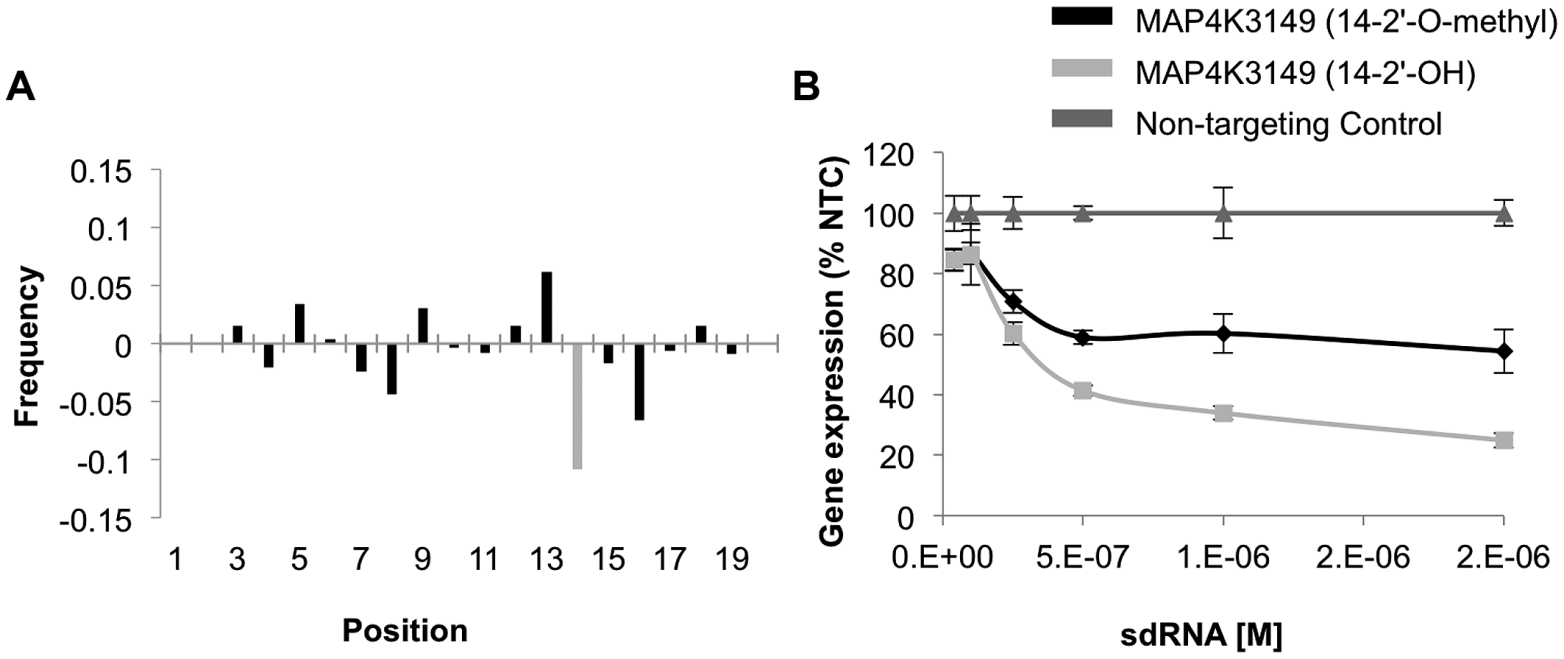
2′-O-methyl modification at position 14 of the antisense strand negatively modulates sdRNA efficacy. (**A**) The frequency of 2′-*O*-methyl modification per position of the antisense strand in functional (defined as < 24% gene expression remaining) versus non-functional (defined as >44% gene expression remaining) sdRNAs. (**B**) The efficacy of sdRNA targeting MAP4K4 with and without 2′-*O*-methyl modification in position 14 of the antisense strand. MAP4K4 expression was analyzed by qPCR in HeLa cells treated with sdRNAs for 72 h (*n* = 3, mean ± SD; one-way ANOVA *P* < 0.001).

All pyrimidines in the antisense strand of the sdRNA compounds used in this study were 2′-fluoro modified. In addition, 156 antisense strands contained at least one additional 2′-*O*-methyl modification, which was introduced to disrupt continuous stretches of five or more non-modified nucleotides. This construction gave us an opportunity to evaluate the tolerance of 2′-*O*-methyl modifications in the antisense strand. In all positions but position 14, 2′-*O*-methyl modification was well tolerated, and sequences including 2′-*O*-methyl modifications were equally distributed between functional and non-functional sdRNA subsets (Figure 6A, Supplementary Figure S3A and B). Out of 19 siRNAs that contained 2′-*O*-methyl modification in position 14, none were functional. This result indicates that 2′-*O*-methyl modification is not well tolerated at position 14 in the context of heavily modified siRNAs. Introduction of a 2′-*O*-methyl modification at position 14 of the functional sdRNA targeting MAP4K4 resulted in a significant loss of efficacy (Figure 6B, Supplementary Figure S3C and D, Supplementary Table S2).

## DISCUSSION

In this study, we performed the first systematic comparison of siRNA prediction algorithms derived from uniform datasets of modified and non-modified siRNA sequences. We developed an algorithm for predicting the efficacy of heavily modified siRNAs that describes a training dataset with 85% predictive power at 40% sensitivity. When tested on two independent datasets, the developed algorithm predicted compound efficacy with 60–80% accuracy at 25% sensitivity. While the training set was generated using a reporter assay, in the two validation sets, sdRNA efficacy was evaluated using qPCR and QuantiGene assays, which have inherently higher noise levels. Thus, the derived algorithm is capable of predicting sdRNA efficacy independent of the methodology used for the measurement of siRNA activity.

There are many ways to derive algorithms predicting siRNA efficacy, most of which produce outputs with similar predictive power (∼60% on validation datasets) (7). Here we developed a linear regression-based algorithm that predicted the efficacy of sdRNAs with an accuracy comparable to other models reported previously (7). The linear regression model used positional base preferences as descriptors and allowed for simple visualization of the major features contributing to functional efficacy. This ability enabled a straightforward connection to the underlying molecular mechanism. In addition, this methodology is easily adaptable for the description of any dataset, can be independently reproduced, and does not require access to advanced mathematical models or exceptional computational power. Application of this algorithm effectively removes the requirement for stochastic screening for the identification of potent compounds, effectively making the algorithm a feasible procedure with limited time constraint that is not experimentally challenging.

Here, we also demonstrated that the functional determinants defining the efficacy of modified and non-modified siRNA are substantially different. Consequently, selection algorithms generated from non-modified siRNAs have low predictive power for modified siRNA compounds and vice versa. In our study, we used a specific class of asymmetric, heavily modified, self-delivering siRNA with a unique modification scaffold. In this scaffold, the functional asymmetry, one of the major determinants of naked siRNA efficacy, is introduced chemically, rendering the thermodynamic bias (the primary determinant for the majority of non-modified siRNA algorithms) non-essential. Since this chemical asymmetry is a feature of many other heavily chemically modified scaffolds (16,44), it is possible that our algorithm may have prediction power on them as well, considering that certain nucleotide preferences are observed near the cleavage site. However, it seems more likely that the positional preference matrix for predicting the efficacy of each class of chemically modified siRNAs will require adjustment for each chemical and/or structural scaffold. Using our approach, a new positional preference matrix can be quickly generated for a specific set of data and included in the original algorithm flow, making it widely adoptable and easily applicable for the prediction of functional siRNA of any class.

The only region where the positional base preferences were similar between non-modified and modified siRNAs was in positions 6–8 of the 20 base siRNA targeting site. Salomon *et al.* have defined the relative contribution of different mechanistic steps in a RISC complex function (45). They identified the rate of product release as one of the major factors limiting overall RISC efficacy. The uniformly observed preference for low GC content at positons 6–8 (5′ to cleavage site) might be contributing to more efficient product release. It is impossible to distinguish if this preference contributes to the first step of RISC loading (passenger strand release), target cleavage, or both. The initial passenger strand release step can occur either through cleavage or dissociation (40). Extensive modification of the sense strand might interfere with cleavage, thus making efficient sense strand dissociation a predominant mechanism for RISC loading.

Additionally, we identified that position 14 of the antisense strand does not tolerate 2′-*O*-methyl modification. This result is consistent with the original observation (25) that bulky modifications were not tolerated in the context of naked siRNAs. A recent Alnylam paper (44) studying the impact of the tolerance of different modifications patterns on 15 target sites also identified position 14 as the most negatively affected by 2′-*O*-methyl modification. The RISC complex crystal structure does not provide a clear explanation for this phenomenon. It is possible that the presence of the 2′-*O*-methyl interferes with the efficiency of the mRNA ‘kinking,’ which was hypothesized to contribute to the positioning of the mRNA into the RISC active center (45). The negative impact of 2′-*O*-methyl in this position is only pronounced in the context of heavily modified siRNA sequences, and modification of position 14 alone had no impact on siRNA efficacy (20).

Another observation derived from this study is the relative importance of high AU preference immediately outside the targeting region for overall sdRNA efficacy. The potential importance of mRNA accessibility around the siRNA targeting site has been reported previously (41–43). According to our data, the relative contribution of the neighboring region flanking at the 3′ end of the RISC binding site is significantly more pronounced than that of the 5′ region, a finding consistent with the observed mechanics of RISC interaction with the mRNA (40). High AU content 3′ to the seed-binding site minimizes the chances of the seed region being hidden in local secondary structure, which would limit initial accessibility. The relative importance of the regions outside the 20-base RISC binding site for overall siRNA efficacy manifested stronger than previously reported (41–43). This result might be related to the method of screening that we used, in which siRNA activity was evaluated in the context of the 3′ UTR of a reporter gene. The effect might be less pronounced for ORFs, where active translation will disrupt the local RNA structure.

In conclusion, here we demonstrated that the critical parameters defining the efficacy of modified and non-modified siRNA differ significantly, and we developed an algorithm for predicting efficacy of heavily modified siRNAs.

## Supplementary Material

Supplementary Data
