# Supplementary material for: Functional features defining the efficacy of cholesterol-conjugated, self-deliverable, chemically modified siRNAs
Source: Nucleic Acids Res. 2018 Aug 29;46(20):10905–16. doi: 10.1093/nar/gky745 (PMC6237813; doi:10.1093/nar/gky745)
Supplement: Supplementary Data [file gky745_supplemental_files.pdf]

Supplementary Table 1. Sequence and functionality information for all sdRNAs. Listed in the table are the 50-nt target sequences containing the flaking regions and the corresponding sdRNA efficacy values, obtained in the functional screenings.

Supplementary Table 2. Sequence and modification pattern of sdRNA in Figure 6. The demonstrated sequences differ only in the chemical modification of position 14 in the antisense strand.

Supplementary Figure 1. Characterization of datasets. (**A**) The distribution of GC content of the siRNA-targeting region of 356 sdRNAs targeting 17 genes. (**B**) The distribution of the GC content of the siRNA-targeting regions of 2385 siRNAs, the efficacy of which is depicted in C. (**C**) The efficacy of 2385 siRNAs evaluated using an eCFP-eYFP dual reporter assay. Data from Huesken et al., 2005. Functionality cutoffs indicated.

Supplementary Figure 2. **Algorithm performance visualized as ROC curves.** Using linear regression analysis (R 3.4.1), the scoring algorithm was generated for positional preference matrices shown in Figure 2A. Black line shows performance of the control algorithm (see Methods). Area under the curve (AUC) for each curve is indicated.

Supplementary Figure 3. 2′-O-methyl modification at position 14 of the antisense strand negatively modulates sdRNA efficacy. (**A**) The frequency of 2′-O-methyl modification per position of the antisense strand in functional (defined as < 24% gene expression remaining) sdRNAs. (**B**) The frequency of 2′-O-methyl modification per position of the antisense strand in non-functional (defined as > 44% gene expression remaining) sdRNAs. (**C, D**) The efficacy of sdRNA targeting MAP4K4 with and without 2′-O-methyl modification in position 14 of the antisense strand (qPCR, 72 hours, n-3, SD, one-way ANOVA $p < 0.05$). Different chemical modification patterns used in C and D, but within the graphs, only the position 14 modification differs.

Supplementary Figure 1

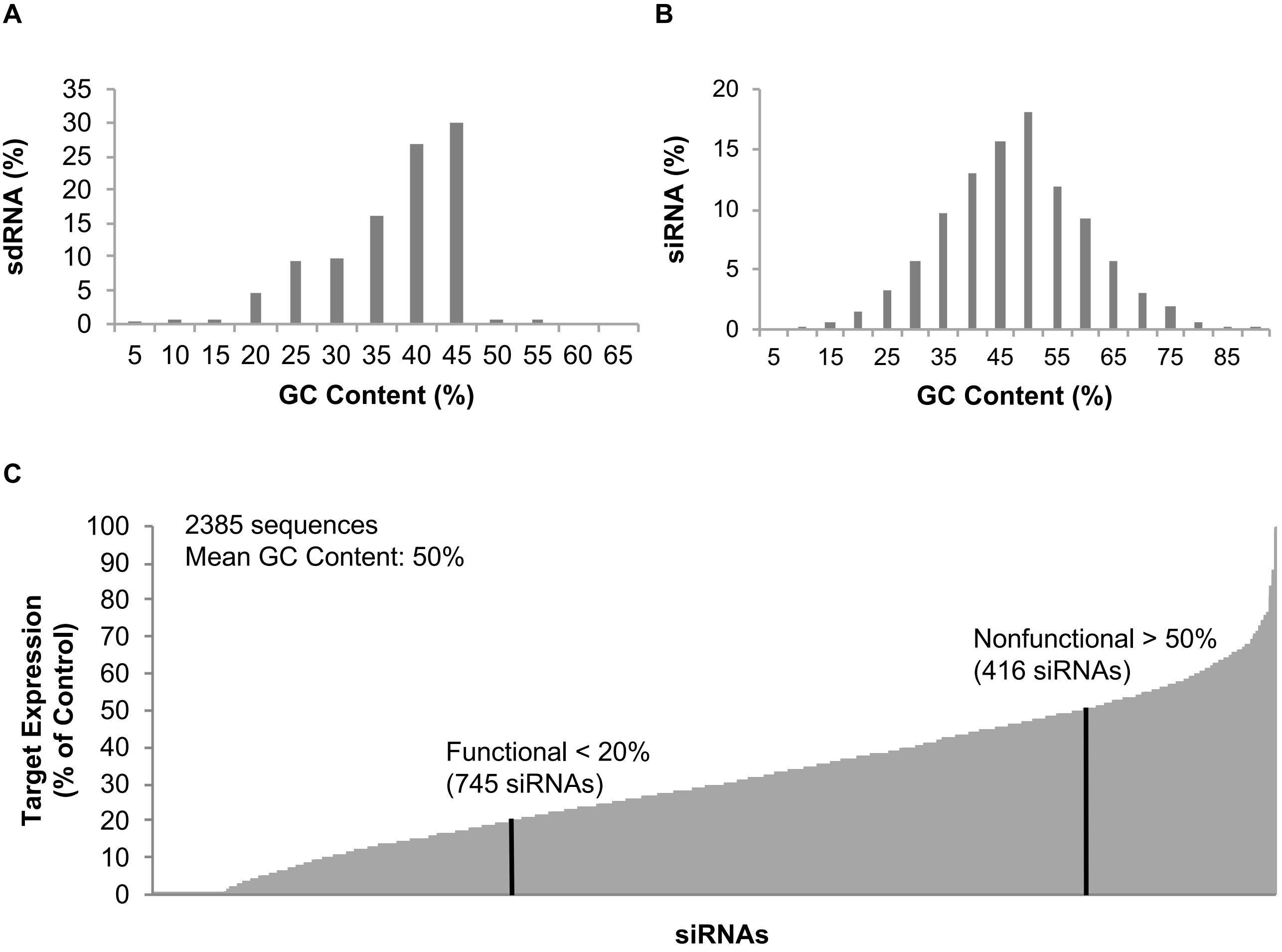

Supplementary Figure 2

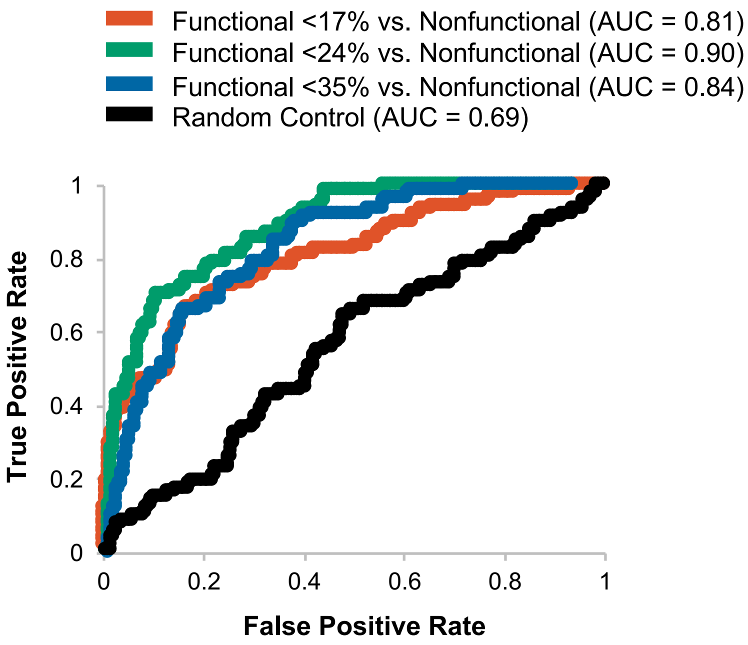

Supplementary Figure 3

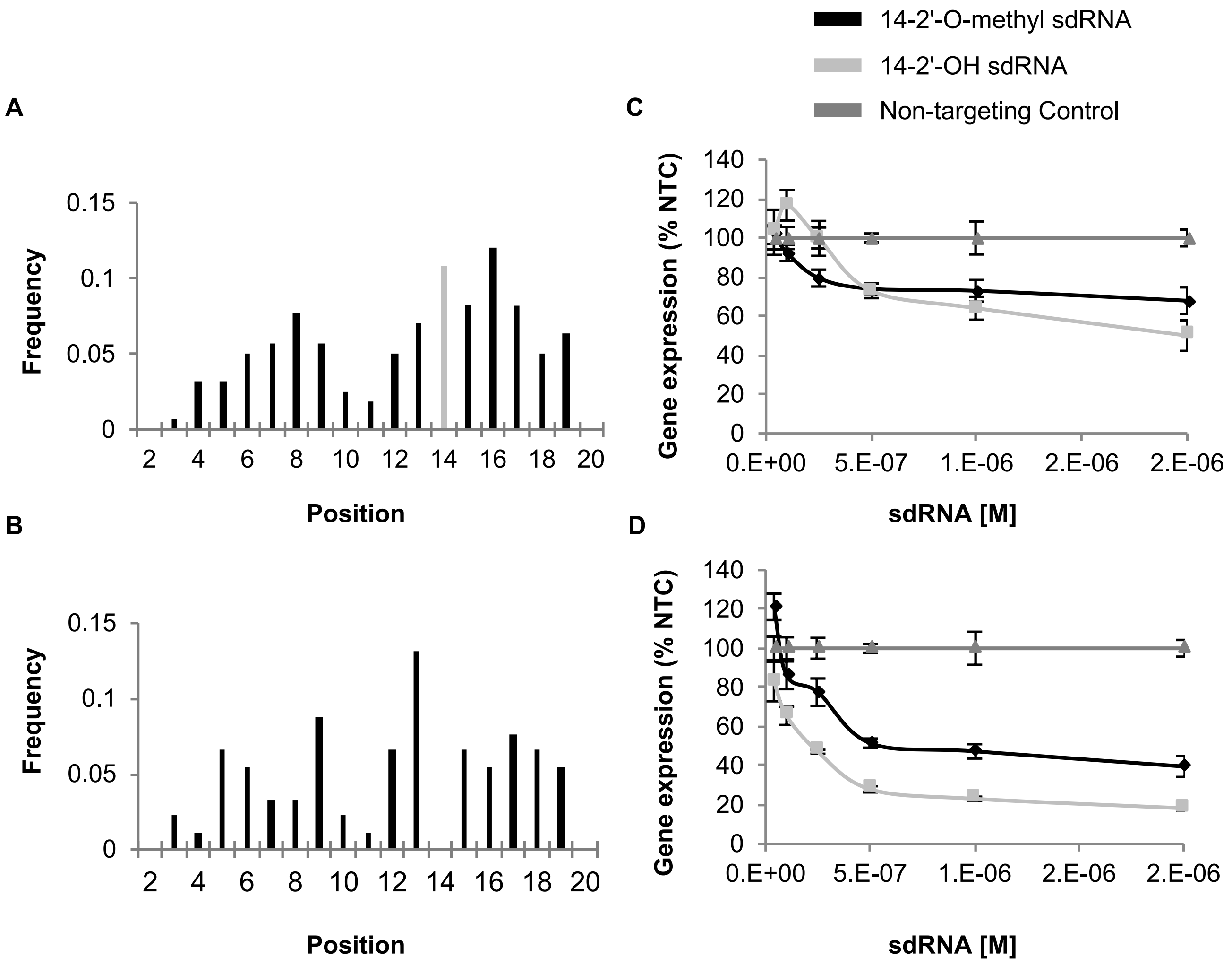

Table of Contents

Supplementary Table 1	S2-S10
Supplementary Table 2	S11

Supplementary Table 1. Sequence and functionality information for all sdRNAs.

ID	5' Region - **siRNA Targeting Region** - 3' Region[1]	Target Expression (% of Control)	Standard Deviation[2]
1000	UUUCCUUGGCUUUAU**GCACAUUAAACAGAUGUGAA**UAUUCGUUUAAACUG	17.10	1.85
1001	AAGAAAUCUCGGAUU**GAUCUAAUGAAGGUAUUUAA**GCAUUUCCACAGCCA	6.50	0.69
1002	CAAGCACAUGGAAGA**UGCUUUGUUUUGUGAGGUAA**UAAACAUGUGAGGAU	3.49	0.25
1003	AUUUGUGGUUAUACU**GUUGGCAUGUAUCUACUUGA**GCUAUUACUUCAGCC	4.61	0.37
1004	AAUGUACUGAAAUUA**AAGGAGGAAGAUAAACAAAA**AUUUAACAAUGCUGA	5.07	0.08
1005	GUUUUUGUGUAUUUG**UUAAUGGUUUGAAUAUAAAC**ACUAUAUGGCAGUGU	5.70	0.31
1006	CCUGGCAACUGAGAA**CAAAGCAACAAGCAACUGAA**UCUCAUUUCUUCAGC	5.79	0.43
1007	AUUCUAGGCGAUCGC**GUGCUCGAUGAGUUAGAAGA**ACUUUGAGCAAGUUA	7.81	1.39
1008	UAUUGAAUUCGGAAC**UGUCCUUUCCUUGGCUUUAU**GCACAUUAAACAGAU	8.21	0.31
1009	AAUUCCAGAACUAGU**GAAAUUAAGGAAAGAAUACU**UAUGAAUUCAAGACC	8.45	0.65
1010	UGUCCUUUCCUUGGC**UUUAUGCACAUUAAACAGAU**GUGAAUAUUCGUUUA	8.69	0.37
1011	CAUCAUCAAAGAGAU**CGUUAGCAGAAACAAAAGGA**GAUAUCAAGAGGAUG	8.93	0.53
1012	UUAAUGUAUAGAUUU**GAAGAAGAGCUGUUUCUGAG**GAGCCUUCAGGAUUA	10.31	0.93
1013	GCUUGCUCUUGUCAU**CUCUUUGAUAUAUUCCUGAA**GGUGCAACAAUCAUU	10.33	0.46
1014	AAGGGAUCCCCCUUC**CCUGUGGUUCUAUUAUAUUA**UAAUUAUAAUUAAAU	10.40	0.34
1015	CGAUGAGUUAGAAGA**ACUUUGAGCAAGUUACUGUG**AGUAUGUAAUACGCA	10.97	3.08
1016	GCUGCCAAGAGUGCU**CGAGAUUUGGACCUAAGAGA**CGGACCUGGACCCCC	11.28	0.30
1017	CAGGAAGCAAGUUUU**UCUGUCAGGAAAACAAUGUU**GGCUUCUGUUGACCU	11.68	0.75
1018	AGGCGCUAUGUAUAU**UAUUAUAGCUACCUGUUAAA**GAAUCACCUGGAUUA	11.74	0.51
1019	AUUGAAUUCGGAACU**GUCCUUUCCUUGGCUUUAUG**CACAUUAAACAGAUG	11.97	0.85
1020	GAAGAUCACCCUCCU**UAAAUAUUUCCGCAAUUACA**UGAGCGAGCACUUGC	12.73	1.17
1021	UAAUUCUAGGCGAUC**GCAUUAUGUCAGAAAUUAAA**AAAUUCUUUAACUUA	12.86	1.66
1022	GUAAUACGCACUGAC**CCAUAUGUCCACCCAUUGAU**GUGAUAUUUUAGCAU	13.07	2.91
1023	CACAUGGAAGAUGCU**UUGUUUUGUGAGGUAAUAAA**CAUGUGAGGAUGAAA	13.07	4.16
1024	GAAGAGCUGUUUCUG**AGGAGCCUUCAGGAUUACAA**GAUUCAAAGUGCGCU	13.28	0.83
1025	UUGUACAGAAUAUUU**CUAUUGAAUUCGGAACUGUC**CUUUCCUUGGCUUUA	13.50	1.77
1026	GACAAGGAUCCCCC**UUCCCUGUGGUUCUAUUAUA**UUAUAAUUAUAAUUA	13.57	1.63
1027	UCGGAACUGUCCUUU**CCUUGGCUUUAUGCACAUUA**AACAGAUGUGAAUAU	13.92	1.26
1028	GGAACUGUCCUUUCC**UUGGCUUUAUGCACAUUAAA**CAGAUGUGAAUAUUC	13.93	0.88
1029	GCAUUGCCCUCAACG**ACCACUUUGUCAAGCUCAUU**UCCUGGUAUGACAAC	14.39	0.01
1030	UAUCCAACAAUUAAU**CGCUUGUAUUACCGAGUAAU**GAUCUUAUUAAACAA	14.51	1.74
1031	GGUGACAUCAAAGUA**GAGUUCUUCCACAAACAGAA**CAAGAUGCUCAAAAA	14.64	0.17
1032	UUUAACUCAAUAUUU**UCCAUGAAAAUGCAACAACA**UGUAUAAUAUUUUUA	14.85	0.53
1033	CCCUCAACGACCACU**UUGUCAAGCUCAUUUCCUGG**UAUGACAACGAAUUU	14.87	1.03
1034	AAUGUCCGUUCGGUU**GGCAGAAGCUAUGAAACGAU**AUGGGCUGAAUACAA	15.13	0.50
1035	GGGCUGCUUUUAACU**CUGGUAAAGUGGAUAUUGUU**GCCAUCAAUGACCCC	15.22	0.15
1036	CCACCAUAUGAAUUG**UACAGAAUAUUUCUAUUGAA**UUCGGAACUGUCCUU	15.34	0.89
1037	GAUAACCGGGAGAUA**GUGAUGAAGUACAUCCAUUA**UAAGCUGUCGCAGAG	15.41	1.14
1038	GAGGCGAACUGUGUG**UGAGAGGUCCUAUGAUUAUG**UCCGGUUAUGUAAAC	16.06	1.80

1039	GAGGCGCUAUGUAUA**UUAUUAUAGCUACCUGUUAA**AGAAUCACCUGGAUU	16.13	0.93
1040	ACAAGGGAUCCCCCU**UCCCUGUGGUUCUAUUAUAU**UAUAAUUAUAAUUAA	16.75	0.85
1041	CUGUCCUUUCCUUGG**CUUUAUGCACAUUAAACAGA**UGUGAAUAUUCGUUU	16.86	0.94
1042	CUUUAACUUACUAAA**AACACUAUCCAACAAUUAAU**CGCUUGUAUUACCGA	17.17	1.96
1043	UGAAUUGUACAGAAU**AUUUCUAUUGAAUUCGGAAC**UGUCCUUUCCUUGGC	17.31	1.96
1044	UGUCAUCUCUUUGAU**AUAUUCCUGAAGGUGCAACA**AUCAUUUGAAAAGGC	17.84	0.55
1045	CAUUGACCUCAACUA**CAUGGUUUACAUGUUCCAAU**AUGAUUCCACCCAUG	18.06	1.04
1046	CGUCAUGGGUGUGAA**CCAUGAGAAGUAUGACAACA**GCCUCAAGAUCAUCA	18.09	1.81
1047	AAUGUUUACUACACU**CGGAUAUUUGAUAUGUGGAU**UUCGAGUCGUCUUAA	18.13	1.38
1048	UUUCGAGUCGUCUUA**AUGUAUAGAUUUGAAGAAGA**GCUGUUUCUGAGGAG	18.16	0.62
1049	ACGACCACUUUGUCA**AGCUCAUUUCCUGGUAUGAC**AACGAAUUUGGCUAC	18.18	0.74
1050	UGUCCACCCAUUGAU**GUGAUAUUUUAGCAUUUUGA**AAGUUGUUGAAACAG	18.27	2.27
1051	GAUUGACAAAUACGA**UUUAUCUAAUUUACACGAAA**UUGCUUCUGGUGGCG	18.28	0.31
1052	CAGGAAAACAAUGUU**GGCUUCUGUUGACCUUUAAA**UGAUUGUCUGAACAC	18.73	2.42
1053	UCACCAGGGCUGCUU**UUAACUCUGGUAAAGUGGAU**AUUGUUGCCAUCAAU	18.93	1.50
1054	UGGAUUUCGAGUCGU**CUUAAUGUAUAGAUUUGAAG**AAGAGCUGUUUCUGA	19.12	0.85
1055	CAUUCAAAAUGAAAA**UAUUUUUAGCACUGACUGAA**GAACUCUUGGGACAA	19.32	2.88
1056	UCCCUCAGCCAUUGC**CUGUGUGUGGUGACAUCAAA**GUAGAGUUCUUCCAC	19.51	0.50
1057	UGACCUCAACUACAU**GGUUUACAUGUUCCAAUAUG**AUUCCACCCAUGGCA	19.52	1.00
1058	AAGUUAAAUUUUAUG**CCUUUUAUUUCUUAAACAAA**UGUAUGAUUACAUCA	19.66	0.44
1059	AUGAUGUAGUAAGGU**UUUUGGAUUCAAAGCAUAAA**AACCAUUACAAGAUA	19.68	6.62
1060	UGUACAGAAUAUUUC**UAUUGAAUUCGGAACUGUCC**UUUCCUUGGCUUUAU	19.87	2.37
1061	GGAGCCGCACCUUGU**CAUGUACCAUCAAUAAAGUA**CCCUGUGCUCAACCA	19.89	1.42
1062	GCUGGUGACAUUUGU**GGUUAUACUGUUGGCAUGUA**UCUACUUGAGCUAUU	19.99	1.75
1063	UUCCGAGAGAAGAAA**UCUCGGAUUGAUCUAAUGAA**GGUAUUUAAGCAUUU	20.16	1.98
1064	CCUCCCCCUCAACCC**CACCAUAUGAAUUGUACAGA**AUAUUUCUAUUGAAU	20.37	0.60
1065	GUGGAAAAUGGAAGU**CUUUGUGAUCAGGAAAUCGA**UAGCAUUUGUAGUAU	20.44	1.53
1066	GUAUGAGACGUUUAU**AGCCGAAAUGAUCUUUUCAA**GUUAAAUUUUAUGCC	20.57	0.74
1067	CAUGUUCGUCAUGGG**UGUGAACCAUGAGAAGUAUG**ACAACAGCCUCAAGA	20.58	1.11
1068	GGUGAAGGUCGGAGU**CAACGGAUUUGGUCGUAUUG**GGCGCCUGGUCACCA	20.66	2.89
1069	UUGACAAGGAUGGAU**GGCUACAUUCUGGAGACAUA**GCUUACUGGGACGAA	21.29	1.03
1070	GACAAAUACGAUUUA**UCUAAUUUACACGAAAUUGC**UUCUGGUGGCGCUCC	21.44	2.36
1071	UAUAUAUAUUUUAAU**UUGAUAGUAUUGUGCAUAGA**GCCACGUAUGUUUUU	21.57	0.38
1072	AUUGUACAGAAUAUU**UCUAUUGAAUUCGGAACUGU**CCUUUCCUUGGCUUU	21.63	2.94
1073	GAACCGCUGGAGAGC**AACUGCAUAAGGCUAUGAAG**AGAUACGCCCUGGUU	22.24	0.87
1074	UAGGGAGCCGCACCU**UGUCAUGUACCAUCAAUAAA**GUACCCUGUGCUCAA	22.55	0.80
1075	UAUUGCUAUGGGAUU**UCCUGCAGAAAGACUUGAAG**GUGUAUACAGGAACA	22.68	0.23
1076	GAUAUUUGAUAUGUG**GAUUUCGAGUCGUCUUAAUG**UAUAGAUUUGAAGAA	22.93	0.88
1077	GGUGUAUACAGGAAC**AAUAUUGAUGAUGUAGUAAG**GUUUUUGGAUUCAAA	23.15	1.04
1078	CAACUACAUGGUUUA**CAUGUUCCAAUAUGAUUCCA**CCCAUGGCAAAUUCC	23.17	2.31
1079	AAAGCACUCUGAUUG**ACAAAUACGAUUUAUCUAAU**UUACACGAAAUUGCU	23.18	1.25
1080	UGAUAACAGCGUGGG**GGUGCUCUUCAAUGACUCAA**CACGCCUCAUCCUCU	23.32	1.44

1081	GGGACAAGGGAUCCC**CCUUCCCUGUGGUUCUAUUA**UAUUAUAAUUAUAAU	23.38	1.76
1082	UUUCUGAGGAGCCUU**CAGGAUUACAAGAUUCAAAG**UGCGCUGCUGGUGCC	23.55	1.56
1083	UACGAUAACCGGGAG**AUAGUGAUGAAGUACAUCCA**UUAUAAGCUGUCGCA	23.57	1.38
1084	UCAUUUCCUGGUAUG**ACAACGAAUUUGGCUACAGC**AACAGGGUGGUGGAC	23.69	0.65
1085	CAUGGGUGUGAACCA**UGAGAAGUAUGACAACAGCC**UCAAGAUCAUCAGCA	23.72	2.67
1086	CUUUUAACUCUGGUA**AAGUGGAUAUUGUUGCCAUC**AAUGACCCCUUCAUU	23.74	1.12
1087	UCAACGACCACUUUG**UCAAGCUCAUUUCCUGGUAU**GACAACGAAUUUGGC	23.82	1.66
1088	AGGGAUCCCCCUUCC**CUGUGGUUCUAUUAUAUUAU**AAUUAUAAUUAAAUA	23.90	0.93
1089	CUUCGCCAAAAGCAC**UCUGAUUGACAAAUACGAUU**UAUCUAAUUUACACG	23.91	0.48
1090	CCAGGGCUGCUUUUA**ACUCUGGUAAAGUGGAUAUU**GUUGCCAUCAAUGAC	23.91	1.62
1091	ACCACCAGCCCCAGC**AAGAGCACAAGAGGAAGAGA**GAGACCCUCACUGCU	24.57	2.65
1092	GCUGGGCAAAGAAGU**CCUUACGGAGUACCUAGAAA**AAUUAGUACAAAGCA	24.63	4.19
1093	ACAAAAAAUGAUCUU**GACAAAGCAAACAAAGACAA**GGCCAACCGAUACUU	24.84	1.49
1094	GAAGGUGAAGGUCGG**AGUCAACGGAUUUGGUCGUA**UUGGGCGCCUGGUCA	25.13	1.71
1095	GAAUUGUACAGAAUA**UUUCUAUUGAAUUCGGAACU**GUCCUUUCCUUGGCU	25.405	1.82
1096	UCAAGCUCAUUUCCU**GGUAUGACAACGAAUUUGGC**UACAGCAACAGGGUG	26.66	0.44
1097	UAUAAAGUCCUUGAU**UCUGUGUGGGUUCAAACACA**UUUCAAAGCUUCAGG	26.79	0.52
1098	CAAUCAUCCAAAAAA**UUAUUAUCAUGGAUUCUAAA**ACGGAUUACCAGGGA	26.85	2.21
1099	CUAUGAAACGAUAUG**GGCUGAAUACAAAUCACAGA**AUCGUCGUAUGCAGU	26.93	1.38
1100	GGAUGCAGCAUUAUG**AUGUGGGUCAAGGAAUUAAG**UUAGGGAAUGGCACA	27.73	1.07
1101	UUGCUUUUAAUUAAA**UGGUCUUGUUAGAAAACAAA**GCUUUUUUGUAUUGG	27.86	4.32
1102	GCCUAGGGAGCCGCA**CCUUGUCAUGUACCAUCAAU**AAAGUACCCUGUGCU	27.91	2.12
1103	CGUGAGUUCCCAUCC**CAACUCCUUGAUGAAGAAGA**UCACCCUCCUUAAAU	28.07	1.36
1104	CUCAAAGACCCUUCG**UUCUUUAACCACGUUCUUGA**AAUCAGGGUCCCCAA	28.47	0.14
1105	GGGUGUGAACCAUGA**GAAGUAUGACAACAGCCUCA**AGAUCAUCAGCAAUG	28.58	0.86
1106	UGUUGGUGGAUGAGC**AAGAGACCUUGCUUAUUAGA**AACAAAGUUUAAACU	28.61	3.52
1107	UUAACUCUGGUAAAG**UGGAUAUUGUUGCCAUCAAU**GACCCCUUCAUUGAC	29.2	0.42
1108	CGAUAACCGGGAGAU**AGUGAUGAAGUACAUCCAUU**AUAAGCUGUCGCAGA	29.30	1.61
1109	GAAACAGGAAGCAAG**UUUUUCUGUCAGGAAAACAA**UGUUGGCUUCUGUUG	29.38	1.65
1110	UGCCAUCAAUGACCC**CUUCAUUGACCUCAACUACA**UGGUUUACAUGUUCC	29.41	0.76
1111	GUCUGACAACCUCUU**GGUGAACCUUAGUACCUAAA**AGGAAAUCUCACCCC	29.63	0.65
1112	GGGGAGGAGGAUGGG**GAGUAGGACAUACCAGCUUA**GAUUUUAAGGUUUUU	29.76	3.76
1113	UUUCCUGGUAUGACA**ACGAAUUUGGCUACAGCAAC**AGGGUGGUGGACCUC	29.81	1.07
1114	AUUCCAUCACGGUUU**UGGAAUGUUUACUACACUCG**GAUAUUUGAUAUGUG	30.00	1.74
1115	GGUGCCCACACUAUU**UAGCUUCUUCGCUAAGAGCA**CUCUCAUCGACAAGU	30.22	7.81
1116	CCUCAACUACAUGGU**UUACAUGUUCCAAUAUGAUU**CCACCCAUGGCAAAU	31.13	0.62
1117	GUUUCUGUGCAGCAU**CAAAUCAGGCGUCCUGAAAA**AUGACAGCACCAUCC	31.19	0.49
1118	AACCUGCCAAAUAUG**AUGACAUCAAGAAGGUGGUG**AAGCAGGCGUCGGAG	31.49	5.80
1119	GAGCAAGUUACUGUG**AGUAUGUAAUACGCACUGAC**CCAUAUGUCCACCCA	31.53	2.10
1120	AUUCUAGGCGAUCGC**ACGUUAUUGCAGAAAUCAAA**UAGAUUCAACAUAGA	31.55	3.50
1121	CUUCAUUGACCUCAA**CUACAUGGUUUACAUGUUCC**AAUAUGAUUCCACCC	31.58	1.41
1122	UAACCGGGAGAUAGU**GAUGAAGUACAUCCAUUAUA**AGCUGUCGCAGAGGG	32.00	3.63

1123	AACCGGGAGAUAGUG**AUGAAGUACAUCCAUUAUAA**GCUGUCGCAGAGGGG	32.01	4.81
1124	UCAACCCCACCAUAU**GAAUUGUACAGAAUAUUUCU**AUUGAAUUCGGAACU	32.21	1.45
1125	CCCAGCCAAACCCUG**UCUGACAACCUCUUGGUGAA**CCUUAGUACCUAAAA	32.21	1.40
1126	GGGCUGGCAUUGCCC**UCAACGACCACUUUGUCAAG**CUCAUUUCCUGGUAU	32.22	2.13
1127	UUGCGCCCGCGAACG**ACAUUUAUAAUGAACGUGAA**UUGCUCAACAGUAUG	32.23	0.64
1128	UCGAAAUGUCCGUUC**GGUUGGCAGAAGCUAUGAAA**CGAUAUGGCUGAAU	32.38	0.48
1129	GCUAUCCAGCUAUUU**UUAUUUGUUUGUGCAUUUGG**GGGGAAUUCAUCUCU	32.58	0.80
1130	GCUGCAAAAGAUCCU**CAACGUGCAAAAGAAGCUAC**CGAUCAUACAAAAGA	32.62	5.83
1131	CUUUCCACGAUUGAA**GUAUUUACUUGCUCAGUAAC**GGUCUUUAUUACAAA	32.94	1.72
1132	UCUAUAUAAAGUCCU**UGAUUCUGUGUGGGUUCAAA**CACAUUUCAAAGCUU	33.28	1.56
1133	UGAAGAGAUACGCCC**UGGUUCCUGGAACAAUUGCU**UUUACAGAUGCACAU	33.79	1.79
1134	GGCCAAGGUCAUCCA**UGACAACUUUGGUAUCGUGG**AAGGACUCAUGACCA	33.81	1.72
1135	CGGAUAUUUGAUAUG**UGGAUUUCGAGUCGUCUUAA**UGUAUAGAUUUGAAG	34.21	0.50
1136	GUUCGUCAUGGGUGU**GAACCAUGAGAAGUAUGACA**ACAGCCUCAAGAUCA	34.64	1.16
1137	GUCUAGAAAAACCUG**CCAAAUAUGAUGACAUCAAG**AAGGUGGUGAAGCAG	34.94	4.70
1138	ACAUCAAAGUAGAGU**UCUUCCACAAACAGAACAAG**AUGCUCAAAAAGGAC	35.01	1.47
1139	GUGUAUUUGUUAAUG**GUUUGAAUAUAAACACUAUA**UGGCAGUGUCUUUCC	35.08	3.22
1140	CUUAAACAAAUGUAU**GAUUACAUCAAGGCUUCAAA**AAUACUCACAUGGCU	35.22	1.09
1141	CCUUCGAUAGGGACA**AGACAAUUGCACUGAUCAUG**AACUCCUCUGGAUCU	35.30	2.01
1142	CAAUGACCCCUUCAU**UGACCUCAACUACAUGGUUU**ACAUGUUCCAAUAUG	35.31	1.52
1143	CUGGAACAAUUGCUU**UUACAGAUGCACAUAUCGAG**GUGGACAUCACUUAC	35.47	2.02
1144	ACCCUGGGAGCUCUC**CUUGGAACCCAUUCCUGAAA**UUAUUUAAAGGGGUU	35.59	3.88
1145	CAGGCACAGCCCCAC**CACAGGACUCAUGUCUCAAU**GCCCACAGUGAGCCC	35.77	2.23
1146	ACCCCUGGCCAAGGU**CAUCCAUGACAACUUUGGUA**UCGUGGAAGGACUCA	35.82	1.03
1147	ACUUUGUCAAGCUCA**UUUCCUGGUAUGACAACGAA**UUUGGCUACAGCAAC	35.85	1.18
1148	GGAUACUGCGAUUUU**AAGUGUUGUUCCAUUCCAUC**ACGGUUUUGGAAUGU	35.96	1.91
1149	GUGAUGAAGUACAUC**CAUUAUAAGCUGUCGCAGAG**GGGCUACGAGUGGGA	36.08	1.84
1150	UGUGAACCAUGAGAA**GUAUGACAACAGCCUCAAGA**UCAUCAGCAAUGCCU	36.31	0.17
1151	UUGUCAAGCUCAUUU**CCUGGUAUGACAACGAAUUU**GGCUACAGCAACAGG	36.70	1.28
1152	UGUGAGGGAUGUUUG**GGAGAUGUAAGAAAUGUUCU**UGCAGUUAAGGGUUA	36.72	1.60
1153	ACUCUCGGACGAGUU**UCUGUGCAGCAUCAAAUCAG**GCGUCCUGAAAAAUG	36.83	1.17
1154	UGAGCUCAAAGACCC**UUCGUUCUUUAACCACGUUC**UUGAAAUCAGGGUCC	36.88	2.39
1155	UCAAGUUAAAUUUUA**UGCCUUUUAUUUCUUAAACA**AAUGUAUGAUUACAU	36.95	0.53
1156	UUAUUGCUAUGGGAU**UUCCUGCAGAAAGACUUGAA**GGUGUAUACAGGAAC	37.28	1.65
1157	CCCUGACAAAAAAUG**AUCUUGACAAAGCAAACAAA**GACAAGGCCAACCGA	37.40	3.23
1158	GGAAGCGACCAACGC**CUUGAUUGACAAGGAUGGAU**GGCUACAUUCUGGAG	37.96	2.68
1159	UCUGGGAUCAAAGCU**AUCUAUAUAAAGUCCUUGAU**UCUGUGUGGGUUCAA	37.98	1.72
1160	AGCACCCCUGGCCAA**GGUCAUCCAUGACAACUUUG**GUAUCGUGGAAGGAC	38.40	2.18
1161	UUGUUAGAAAACAAA**GCUUUUUUGUAUUGGUUAAA**CUCGAGUGGUGAUUG	38.52	1.96
1162	GUUAAGGGUUAGUUU**ACAAUCAGCCACAUUCUAGG**UAGGGGCCCACUUCA	38.60	5.11
1163	CAUCUCUUGUAUAUG**AUGAUCUGGAUCCACCAAGA**CUUGUUUUAUGCUCA	38.66	1.58
1164	UCACAUUCUAUUCCU**GAAAUUCCAGAACUAGUGAA**AUUAAGGAAAGAAUA	38.80	2.04

1165	GCUGAGAACGGGAAG**CUUGUCAUCAAUGGAAAUCC**CAUCACCAUCUUCCA	38.86	1.51
1166	GGUACGAUAACCGGG**AGAUAGUGAUGAAGUACAUC**CAUUAUAAGCUGUCG	38.93	2.99
1167	UAUUGUUUUCAGGGG**GCCUAUGGCUAAAUCAAAUU**AACCUACCCCGCAUA	38.93	1.60
1168	UUGCCCUCAACGACC**ACUUUGUCAAGCUCAUUUCC**UGGUAUGACAACGAA	38.95	1.79
1169	ACAUAUCGAGGUGGA**CAUCACUUACGCUGAGUACU**UCGAAAUGUCCGUUC	39.11	1.37
1170	CGAGGUGGACAUCAC**UUACGCUGAGUACUUCGAAA**UGUCCGUUCGGUUGG	39.23	0.54
1171	GACAACAUCGCCCUG**UGGAUGACUGAGUACCUGAA**CCGGCACCUGCACAC	39.33	1.19
1172	CAUCACGGUUUUGGA**AUGUUUACUACACUCGGAUA**UUUGAUAUGUGGAUU	39.39	1.77
1173	AUUUGAUAUGUGGAU**UUCGAGUCGUCUUAAUGUAU**AGAUUUGAAGAAGAG	39.57	1.31
1174	CAUAUGAAUUGUACA**GAAUAUUUCUAUUGAAUUCG**GAACUGUCCUUUCCU	39.61	1.39
1175	AAAAGAGAUCGUGGA**UUACGUCGCCAGUCAAGUAA**CAACCGCGAAAAAGU	39.63	1.58
1176	CUGUUGACCUUUAAA**UGAUUGUCUGAACACAUAAA**CUCGAGUGGUGAUUG	39.73	4.47
1177	ACUUAGACUUGACCU**AUAUUUAUCCAAAUAUUAUU**GCUAUGGGAUUUCCU	39.74	1.82
1178	GAUUCAAAGCAUAAA**AACCAUUACAAGAUAUACAA**UCUAUGUGCUGAGAG	40.00	2.34
1179	CUUAGCACCCCUGGC**CAAGGUCAUCCAUGACAACU**UUGGUAUCGUGGAAG	40.44	1.75
1180	AGGACUCUCUUUUUC**CUCUUCCUGGUAGUUGUAAU**GACCACUCCCAUAAG	40.73	1.68
1181	UCAACAUAGACUACA**UUCCAGAUGGAGGAAAGGAA**ACCUGUUCUGACCCA	40.73	3.61
1182	GGUGAAGCUAAUCUG**GAAAUGGAGGAACCAGAAGA**AUCAUUGAACACUCU	40.74	1.41
1183	ACCCCUAACAGUGCC**AGAAUAAGGCAUUUCUCUAU**UGUUUUCAGGGGGCC	40.74	0.97
1184	GUCAUCCAUGACAAC**UUUGGUAUCGUGGAAGGACU**CAUGACCACAGUCCA	40.92	2.90
1185	UGUUUUCAGGGGGCC**UAUGGCUAAAUCAAAUUAAC**CUACCCCGCAUAGGG	41.15	2.41
1186	GUUGCGCGGAGGAGU**UGUGUUUGUGGACGAAGUAC**CGAAAGGUCUUACCG	41.16	0.81
1187	CUCUAACUUCAAGGC**CCAUAUCUGUGAAAUGCUGG**CAUUUGCACCUACCU	41.22	1.58
1188	AGCUCAUUUCCUGGU**AUGACAACGAAUUUGGCUAC**AGCAACAGGGUGGUG	41.32	1.46
1189	UCUGAUUGACAAAUA**CGAUUUAUCUAAUUUACACG**AAAUUGCUUCUGGUG	42.12	1.67
1190	GGACUCUCUUUUUCC**UCUUCCUGGUAGUUGUAAUG**ACCACUCCCAUAAGG	42.19	1.33
1191	CCCCUUCAUUGACCU**CAACUACAUGGUUUACAUGU**UCCAAUAUGAUUCCA	42.47	1.17
1192	CUUCUUCGCCAAAAG**CACUCUGAUUGACAAAUACG**AUUUAUCUAAUUUAC	42.74	1.67
1193	UCCUAUGAUUAUGUC**CGGUUAUGUAAACAAUCCGG**AAGCGACCAACGCCU	43.01	1.29
1194	UUAGCAUUUUGAAAG**UUGUUGAAACAGGAAGCAAG**UUUUUCUGUCAGGAA	43.26	2.02
1195	UCAAAGACCCUUCGU**UCUUUAACCACGUUCUUGAA**AUCAGGGUCCCCAAC	43.39	2.43
1196	UAGAAAAACCUGCCA**AAUAUGAUGACAUCAAGAAG**GUGGUGAAGCAGGCG	43.74	2.74
1197	GUUCUUGCAGUUAAG**GGUUAGUUUACAAUCAGCCA**CAUUCUAGGUAGGGG	43.74	2.99
1198	GAACCAUGAGAAGUA**UGACAACAGCCUCAAGAUCA**UCAGCAAUGCCUCCU	43.81	1.54
1199	UUAGCACUGACUGAA**GAACUCUUGGGACAAAGGAU**AUAUUUUUUAAAUCA	44.10	1.17
1200	AGUGGCAUCUGUUUU**GUAGGCUACAAGAACUAUCG**GUACCGUGCUGGCUU	44.34	1.44
1201	GUGCGUCAGAAGCAC**CCAGGACUUCCAUUUGCUUU**GUCCCGGGGCUCCAC	44.38	3.12
1202	CAUCAAUGACCCCUU**CAUUGACCUCAACUACAUGG**UUUACAUGUUCCAAU	44.56	0.59
1203	CCUGCCGUCUAGAAA**AACCUGCCAAAUAUGAUGAC**AUCAAGAAGGUGGUG	44.79	3.17
1204	GAGCUGUUUCUGAGG**AGCCUUCAGGAUUACAAGAU**UCAAAGUGCGCUGCU	44.84	3.30
1205	UGUUUGGGAGAUGUA**AGAAAUGUUCUUGCAGUUAA**GGGUUAGUUUACAAU	45.29	5.74
1206	UGACCCCUUCAUUGA**CCUCAACUACAUGGUUUACA**UGUUCCAAUAUGAUU	45.54	1.84

1207	GUUUGGGAGAUGUAA**GAAAUGUUCUUGCAGUUAAG**GGUUAGUUUACAAUC	45.58	4.45
1208	CUGUUGGUGGAUGAG**CAAGAGACCUUGCUUAUUAG**AAACAAAGUUUAAAC	45.63	2.95
1209	CCUACCCCGCAUAGG**GGCUGGACUCUACAAAUAGA**ACUUCACCCAAGGGG	45.96	1.93
1210	GAUUCCACCCAUGGC**AAAUUCCAUGGCACCGUCAA**GGCUGAGAACGGGAA	46.07	3.31
1211	AAAAACCUGCCAAAU**AUGAUGACAUCAAGAAGGUG**GUGAAGCAGGCGUCG	46.18	9.84
1212	GUGAACUGGGGGAGG**AUUGUGGCCUUCUUUGAGUU**CGGUGGGGUCAUGUG	46.19	1.14
1213	GCCGUCUAGAAAAAC**CUGCCAAAUAUGAUGACAUC**AAGAAGGUGGUGAAG	46.33	4.34
1214	UUUUAACUCAAUAUU**UUCCAUGAAAAUGCAACAAC**AUGUAUAAUAUUUUU	46.48	0.76
1215	GGGAGGAGGAUGGGG**AGUAGGACAUACCAGCUUAG**AUUUUAAGGUUUUUA	46.51	4.49
1216	CGCCAAAAGCACUCU**GAUUGACAAAUACGAUUUAU**CUAAUUUACACGAAA	47.07	1.32
1217	AAUGAAGGUAUUUAA**GCAUUUCCACAGCCAUUGAU**UGAAUGCCGCAAUCU	47.47	6.28
1218	CCACCCAUUGAUGUG**AUAUUUUAGCAUUUUGAAAG**UUGUUGAAACAGGAA	47.70	3.03
1219	UAUUAAACAAUCAAA**CUCUUUCACUGUUUUAUAAA**AAAUUUUGCUUUUAA	47.96	0.41
1220	AAGGCUGAGAACGGG**AAGCUUGUCAUCAAUGGAAA**UCCCAUCACCAUCUU	48.27	5.06
1221	UAGUGAUGAAGUACA**UCCAUUAUAAGCUGUCGCAG**AGGGGCUACGAGUGG	48.45	1.24
1222	GGUGAACUGGGGGAG**GAUUGUGGCCUUCUUUGAGU**UCGGUGGGGUCAUGU	48.60	1.45
1223	GUAUUACCGAGUAAU**GAUCUUAUUAAACAAUCAAA**CUCUUUCACUGUUUU	48.84	4.54
1224	GUUUUUGGUAUUGAU**GUAGUGUGUGUCCUACCAAU**CUCUAAUAGACAUAA	48.96	2.06
1225	GAAGAAGCAAUUGGA**UGCUUCUUUCCACGAUUGAA**GUAUUUACUUGCUCA	49.08	1.31
1226	GCCUGACUCAGACUG**ACAUUCUCCACUUCUUGUUC**CCCACUGACAGCCUC	49.24	0.86
1227	GGUGUGAGCAGUGUC**AGAGUGUGGUAAAGCCUGAU**AUCGUGUUUUUCGGU	49.42	1.70
1228	CCUGGGAGCUCUCCU**UGGAACCCAUUCCUGAAAUU**AUUUAAAGGGGUUGG	49.51	1.59
1229	CCCUGUUGGUCGGUG**GGUUGGUAGUUUCUACAGUU**GGGCAGCUGGUUAGG	49.68	0.97
1230	UCGGCCCAGCCUCCU**GGUGGAGAAGAUCAAUCUAU**UUGCCAUGUUUGGCA	49.75	1.67
1231	UCCACAGCCAUUGAU**UGAAUGCCGCAAUCUAUACA**AACAUGUCUAAAAUU	49.77	6.92
1232	ACCACUUUGUCAAGC**UCAUUUCCUGGUAUGACAAC**GAAUUUGGCUACAGC	49.95	2.49
1233	GCGGAGGAGUUGUGU**UUGUGGACGAAGUACCGAAA**GGUCUUACCGGAAAA	50.27	1.37
1234	AGGGUCAACAUCUUU**UACAUUCUGCAAGCACAUCU**GCAUUUUCACCCCAC	50.33	2.74
1235	UGAGCAGACGGAGUA**UGCCACCAUUGUCUUUCCUA**GCGGAAUGGGCACCU	50.48	2.75
1236	CAAGGUCAUCCAUGA**CAACUUUGGUAUCGUGGAAG**GACUCAUGACCACAG	50.52	3.88
1237	UGGAUGAGCAAGAGA**CCUUGCUUAUUAGAAACAAA**GUUUAAACUGGUGAU	51.16	2.08
1238	UUCUGACCCAUCAUA**AGUUUGAAGAAGCAAUUGGA**UGCUUCUUUCCACGA	51.25	1.95
1239	GAUGGAGGAAAGGAA**ACCUGUUCUGACCCAUCAUA**AGUUUGAAGAAGCAA	51.33	4.05
1240	AUUGUUUUCAGGGGG**CCUAUGGCUAAAUCAAAUUA**ACCUACCCCGCAUAG	51.33	1.68
1241	GGAUUUUUAUGGGGA**AGUAAGGACCAGAGAUAAAA**AGGGAGUAACUAUUC	51.43	2.24
1242	CUGCUUUUAACUCUG**GUAAAGUGGAUAUUGUUGCC**AUCAAUGACCCCUUC	51.56	4.64
1243	ACUCUGGUAAAGUGG**AUAUUGUUGCCAUCAAUGAC**CCCUUCAUUGACCUC	52.10	0.62
1244	CCUGGUAUGACAACG**AAUUUGGCUACAGCAACAGG**GUGGUGGACCUCAUG	52.27	1.36
1245	ACUCCCAGGUGUGAG**CAGUGUCAGAGUGUGGUAAA**GCCUGAUAUCGUGUU	52.37	2.80
1246	UCUUAAACAAAUGUA**UGAUUACAUCAAGGCUUCAA**AAAUACUCACAUGGC	52.41	4.28
1247	CCCCUUCCCUGUGGU**UCUAUUAUAUUAUAAUUAUA**AUUAAAUAUGAGAGC	52.74	4.62
1248	CCCCUCAGCCGUGCC**UGUGUUCUCUGUGGACUAUG**GGGAGCUGGAUUUCC	52.96	1.46

1249	UGUGUGUGAGAGGUC**CUAUGAUUAUGUCCGGUUAU**GUAAACAAUCCGGAA	53.06	1.50
1250	CUGCCAAAUAUGAUG**ACAUCAAGAAGGUGGUGAAG**CAGGCGUCGGAGGGC	53.20	4.69
1251	GGGGCCUCUAACCAC**CAUAGCCUCUAACCACCAUA**GCCUCUAACCACCAU	53.48	1.54
1252	AGAGUGCAUUGUGAG**GGUUAAUGAAAUAAUGUACA**UCUGGCCUUGAAACC	53.59	2.67
1253	UUACAAAGUAUUAGA**ACCUUUAAGAAGUUAAUGAA**CUCGAGUGGUGAUUG	54.25	5.24
1254	AUUGCAGAAAUCAAA**UAGAUUCAACAUAGACUACA**UUCCAGAUGGAGGAA	54.27	3.99
1255	GAUUUCGAGUCGUCU**UAAUGUAUAGAUUUGAAGAA**GAGCUGUUUCUGAGG	55.31	5.77
1256	AGGUGGCAUGGAUUU**UGACUCCAAGAAGGCUUACA**GGGACGUGGCCUGGC	55.36	3.47
1257	UCAUGAACUCCUCUG**GAUCUACUGGUCUGCCUAAA**GGUGUCGCUCUGCCU	55.99	0.00
1258	GAUAGUGAUGAAGUA**CAUCCAUUAUAAGCUGUCGC**AGAGGGGCUACGAGU	56.01	0.15
1259	CCUGGCCAAGGUCAU**CCAUGACAACUUUGGUAUCG**UGGAAGGACUCAUGA	56.25	2.65
1260	CCUUAAAUAUUUCCG**CAAUUACAUGAGCGAGCACU**UGCUGAAGGCAGGUG	56.90	5.78
1261	CUGAAUACAAAUCAC**AGAAUCGUCGUAUGCAGUGA**AAACUCUCUUCAAUU	56.90	0.93
1262	CAAAACCCCUAACAG**UGCCAGAAUAAGGCAUUUCU**CUAUUGUUUUCAGGG	56.94	0.61
1263	GUGGAUGAGCAAGAG**ACCUUGCUUAUUAGAAACAA**AGUUUAAACUGGUGA	57.03	7.00
1264	CGAACGACAUUUAUA**AUGAACGUGAAUUGCUCAAC**AGUAUGGGCAUUUCG	57.08	3.29
1265	ACUCGACGCAAGAAA**AAUCAGAGAGAUCCUCAUAA**AGGCCAAGAAGGGCG	57.43	3.55
1266	UGACAUCAAAGUAGA**GUUCUUCCACAAACAGAACA**AGAUGCUCAAAAAGG	57.87	1.08
1267	GAGGGUUAAUGAAAU**AAUGUACAUCUGGCCUUGAA**ACCACCUUUUAUUAC	58.46	2.03
1268	AGAGGUCCUAUGAUU**AUGUCCGGUUAUGUAAACAA**UCCGGAAGCGACCAA	59.61	1.64
1269	GAAUACAAAUCACAG**AAUCGUCGUAUGCAGUGAAA**ACUCUCUUCAAUUCU	59.82	0.61
1270	CCAUGAGAAGUAUGA**CAACAGCCUCAAGAUCAUCA**GCAAUGCCUCCUGCA	61.07	5.80
1271	UCGUAUGCAGUGAAA**ACUCUCUUCAAUUCUUUAUG**CCGGUGUUGGGCGCG	61.43	1.71
1272	AGUGAUGAAGUACAU**CCAUUAUAAGCUGUCGCAGA**GGGGCUACGAGUGGG	61.94	2.53
1273	GUGAGAGGUCCUAUG**AUUAUGUCCGGUUAUGUAAA**CAAUCCGGAAGCGAC	61.97	1.73
1274	AAUAAAACAAAAAAA**AAAUUUACCAAAAGAGAAAU**UUGUUUUAUUUAUUU	62.59	3.71
1275	UAUGAAACGAUAUGG**GCUGAAUACAAAUCACAGAA**UCGUCGUAUGCAGUG	63.05	1.90
1276	UGGAGAGGCAGAGAU**GGACUUCCUGAGGAAUUUAU**UCACCCAGACCCUGG	63.35	1.34
1277	AGAAGUAUGACAACA**GCCUCAAGAUCAUCAGCAAU**GCCUCCUGCACCACC	63.51	3.76
1278	CAAGGAUAUGGGCUC**ACUGAGACUACAUCAGCUAU**UCUGAUUACACCCGA	64.03	1.48
1279	GGGGUGAACUGGGGG**AGGAUUGUGGCCUUCUUUGA**GUUCGGUGGGGUCAU	64.40	0.62
1280	CUUCCUGGCCUUUGG**CUUUGUGCUCAUCACCUUCA**GCUGCCACUUCUAUG	64.40	1.86
1281	UGUGAGCAGUGUCAG**AGUGUGGUAAAGCCUGAUAU**CGUGUUUUUCGGUGA	64.76	0.77
1282	UUGUUUUCAGGGGGC**CUAUGGCUAAAUCAAAUUAA**CCUACCCCGCAUAGG	64.81	0.22
1283	GGAGCUAUUCUUGCG**CAGCUUGCAAGACUAUAAGA**UUCAAUCUGCCCUGC	65.06	7.75
1284	CCGGGAGAUAGUGAUGAAGUACAUCCAUUAUAAGCUGUCGCAGAGGGGCU	65.16	5.14
1285	GGGAUGCAGCAUUAU**GAUGUGGGUCAAGGAAUUAA**GUUAGGGAAUGGCAC	65.23	6.27
1286	CUUUUACAGAUGCAC**AUAUCGAGGUGGACAUCACU**UACGCUGAGUACUUC	65.24	1.42
1287	AUCGGCCCAGCCUCC**UGGUGGAGAAGAUCAAUCUA**UUUGCCAUGUUUGGC	65.52	5.43
1288	UGAUGAAGUACAUCC**AUUAUAAGCUGUCGCAGAGG**GGCUACGAGUGGGAU	65.62	1.88
1289	CAAAGACCCUUCGUU**CUUUAACCACGUUCUUGAAA**UCAGGGUCCCCAACU	65.82	1.18
1290	UUUUGGAAUGUUUAC**UACACUCGGAUAUUUGAUAU**GUGGAUUUCGAGUCG	66.08	5.94

1291	CAGGACUCUCUUUUU**CCUCUUCCUGGUAGUUGUAA**UGACCACUCCCAUAA	66.34	2.65
1292	AUUCUAGGCGAUCGC**GUGGUUUCCGAGAGAAGAAA**UCUCGGAUUGAUCUA	66.54	1.82
1293	CUGCAGGCCUAGAGA**AGUUUCAGGGAAGGUCAGAA**GAGCUCCUGGCUGUG	66.97	7.95
1294	AUUUUUGGCAAUCAA**AUCAUUCCGGAUACUGCGAU**UUUAAGUGUUGUUCC	67.14	3.64
1295	UCGUACAAGUGUCUG**AAGGUGCUGGAGAACAUUAA**AAGCAAUGGGCAGGG	67.28	3.85
1296	UUUCAAGUUAAAUUU**UAUGCCUUUUAUUUCUUAAA**CAAAUGUAUGAUUAC	67.93	1.24
1297	GAGAUAGUGAUGAAG**UACAUCCAUUAUAAGCUGUC**GCAGAGGGGCUACGA	68.37	0.61
1298	CAAGACCGACUACCA**GGGCUUCCAAAGCAUGUACA**CCUUCGUGACUUCCC	69.14	10.06
1299	UACUGCGAUUUUAAG**UGUUGUUCCAUUCCAUCACG**GUUUUGGAAUGUUUA	69.55	2.36
1300	CUGUGAAAUGCUGGC**AUUUGCACCUACCUCACAGA**GUGCAUUGUGAGGGU	69.84	7.32
1301	GAGUGGCAUCUGUUU**UGUAGGCUACAAGAACUAUC**GGUACCGUGCUGGCU	70.58	3.59
1302	CUCCGUGAGUGGCAU**CUGUUUUGUAGGCUACAAGA**ACUAUCGGUACCGUG	70.93	5.02
1303	AGUGUUUAUUGUGUC**AUUUGUCCUUUGUCUAGGAG**AGGACAGGACUCUCU	71.13	8.63
1304	GCUGGCAUUUGCACC**UACCUCACAGAGUGCAUUGU**GAGGGUUAAUGAAAU	71.63	2.22
1305	GGAACCGCUGGAGAG**CAACUGCAUAAGGCUAUGAA**GAGAUACGCCCUGGU	72.28	0.62
1306	UACUUGCUCAGUAAC**GGUCUUUAUUACAAAGUAUU**AGAACCUUUAAGAAG	73.01	4.02
1307	GGAGAUAGUGAUGAA**GUACAUCCAUUAUAAGCUGU**CGCAGAGGGGCUACG	73.87	4.72
1308	AGUAUGACAACAGCC**UCAAGAUCAUCAGCAAUGCC**UCCUGCACCACCAAC	73.88	3.17
1309	CGGGAGAUAGUGAUG**AAGUACAUCCAUUAUAAGCU**GUCGCAGAGGGGCUA	74.29	7.70
1310	ACAAGCAACUGAAUC**UCAUUUCUUCAGCUUGAAGA**AGUGAUCUUGGCCAA	74.62	2.19
1311	CAUCGUUGACCGCCU**GAAGUCUCUGAUUAAGUACA**AAGGCUAUCAGGUGG	75.04	1.22
1312	CCUCUUCCUGGUAGU**UGUAAUGACCACUCCCAUAA**GGGCUAUAACUGUUC	75.10	15.33
1313	AGGGUUAAUGAAAUA**AUGUACAUCUGGCCUUGAAA**CCACCUUUUAUUACA	75.99	3.29
1314	CAAAGAGGCGAACUG**UGUGUGAGAGGUCCUAUGAU**UAUGUCCGGUUAUGU	76.65	3.10
1315	UGAUGUAGUAAGGUU**UUUGGAUUCAAAGCAUAAAA**ACCAUUACAAGAUAU	78.21	1.45
1316	GAUAUGUGGAUUUCG**AGUCGUCUUAAUGUAUAGAU**UUGAAGAAGAGCUGU	79.54	2.99
1317	ACUGGGCACAGUGAU**GAUGAGCCCAAGAGAAUCAA**GAAGAGCAAGAUGAU	80.23	4.94
1318	AUGUCAGAAAUUAAA**AAAUUCUUUAACUUACUAAA**AACACUAUCCAACAA	80.72	4.18
1319	GGGAUCCCCCUUCCC**UGUGGUUCUAUUAUAUUAUA**AUUAUAAUUAAAUAU	81.30	4.22
1320	UGUCUAGUCAUUUUC**AUAGUGCCCCACAUUCAAGA**ACCAGACAACAGGAU	81.58	4.64
1321	UAAAUUUUAUGCCUU**UUAUUUCUUAAACAAAUGUA**UGAUUACAUCAAGGC	82.26	1.01
1322	GGGAGAUAGUGAUGA**AGUACAUCCAUUAUAAGCUG**UCGCAGAGGGGCUAC	82.74	5.35
1323	CAGAGGGGUCAUGAC**UCUGUUCUCCAUCAAGAGCA**ACCACCCUGGGCUUC	83.10	11.76
1324	CGUCAUAGAAGAAUA**GAUUAGAAACGUCCCACAAG**AGUAAGUGUGCUUUG	83.13	5.78
1325	UCUAUGUGCUGAGAG**ACAUUAUGACACCGCCAAAU**UUAACUGCAGAGUUG	84.53	7.10
1326	GUCUAAAAUUUUAAG**GUUUUCAUUCAAAAUGAAAA**UAUUUUUAGCACUGA	84.61	11.22
1327	CGUGAGUGGCAUCUG**UUUUGUAGGCUACAAGAACU**AUCGGUACCGUGCUG	84.82	5.63
1328	GCUCAACAUGGGGCC**CGGGUUCCAAAGUUUGCAAA**GUUGGGAGUCGAGGG	85.29	3.75
1329	CAUCGUGGACCGGCU**GAAGAGCCUGAUCAAAUACA**AGGGCUACCAGGUAG	85.59	10.39
1330	GCCGCAAUCUAUACA**AACAUGUCUAAAAUUUUAAG**GUUUUCAUUCAAAAU	86.86	2.30
1331	CCUGCAGGCCUAGAG**AAGUUUCAGGGAAGGUCAGA**AGAGCUCCUGGCUGU	87.60	2.91
1332	UCACUGUUUUAUAAA**AAAUUUUGCUUUUAAUUAAA**UGGUCUUGUUAGAAA	87.73	10.46

1333	CCUGUCCUGUGUCAU**CAUCUUUGUCAUUGUGUACU**AUGCCUUGAUGGCUG	87.81	10.74
1334	UGAACUGGGGGAGGA**UUGUGGCCUUCUUUGAGUUC**GGUGGGGUCAUGUGU	87.88	0.59
1335	UUGCUCAGUAACGGU**CUUUAUUACAAAGUAUUAGA**ACCUUUAAGAAGUUA	88.19	12.32
1336	UUUGUGCAUUUGGGG**GGAAUUCAUCUCUCUUUAAU**AUAAAGUUGGAUGCG	88.20	4.50
1337	AUAGUGAUGAAGUAC**AUCCAUUAUAAGCUGUCGCA**GAGGGGCUACGAGUG	90.70	1.38
1338	GGUGGAGAAGAUCAA**UCUAUUUGCCAUGUUUGGCA**CUGGCAUUGCCAUGA	91.39	9.76
1339	UGUCAUCAUCUUUGU**CAUUGUGUACUAUGCCUUGA**UGGCUGGAGUAGUCU	91.85	5.13
1340	AGUGAAAACUCUCUU**CAAUUCUUUAUGCCGGUGUU**GGGCGCGUUAUUUAU	92.53	10.73
1341	GCGGUGGUGUUGUGU**UCGUGGACGAGGUGCCUAAA**GGACUGACCGGCAAG	94.38	8.92
1342	CCUGAAGUCUCUGAU**UAAGUACAAAGGCUAUCAGG**UGGCUCCCGCUGAAU	94.69	1.53
1343	AUGAGGGCUGUUUGC**CCUUUUCUGUGCCAAUGAGU**GCCCUUUUCUGGCAC	95.21	7.33
1344	CUUGGGACAAAGGAU**AUAUUUUUUAAAUCAUUAUG**CUCGAGUGGUGAUUG	95.73	6.53
1345	AAAGAUCAUCAUCAU**GGAUAGCAAGACCGACUACC**AGGGCUUCCAAAGCA	96.63	16.25
1346	GAUGGACCUGUUGCG**GGUUUGGCUUUUGACCUCAA**UGAACCCUCAGCUGA	97.51	4.08
1347	CUUCGAGGAGGAGCU**AUUCUUGCGCAGCUUGCAAG**ACUAUAAGAUUCAAU	101.21	4.07
1348	CAUUUGGGGGGAAUU**CAUCUCUCUUUAAUAUAAAG**UUGGAUGCGGAACCC	102.25	4.03
1349	CAUCAGAGGGGUCAU**GACUCUGUUCUCCAUCAAGA**GCAACCACCCUGGGC	103.05	1.77
1350	ACAUAUCGAGGUGGA**CAUUACCUACGCCGAGUACU**UCGAGAUGAGCGUUC	106.40	15.37
1351	UUCGAGAUGAGCGUU**CGGCUGGCAGAAGCUAUGAA**GCGCUAUGGGCUGAA	109.21	8.64
1352	GCUCUCCUUGGAACC**CAUUCCUGAAAUUAUUUAAA**GGGGUUGGCCGGGCU	109.88	8.13
1353	GAGCUCUCCUUGGAA**CCCAUUCCUGAAAUUAUUUA**AAGGGGUUGGCCGGG	113.42	1.88
1354	UCUCUCCAAAUUUUA**AGGUGAAGUUAUACUUCACA**AAAACAGUAGAGGAG	116.70	8.50
1355	GCAGGCCUAGAGAAG**UUUCAGGGAAGGUCAGAAGA**GCUCCUGGCUGUGGU	119.88	2.99

[1] Shown are mRNA sequences containing 20-nucleotide target sites (in bold) and the flanking regions.
[2] N=3.

Supplementary Table 2. Sequence and modification pattern of sdRNAs in Figure 6.

Name	Sequence[1,2]
MAP4K3149 (14-2'-O-methyl)	P.mU.mA.mG.mA.fC.fU.fU.fC.fC.mA.mC#mA#mG#**mA**#mA#fC#fU#fC#fU
MAP4K3149 (14-2'-OH)	P.mU.mA.mG.mA.fC.fU.fU.fC.fC.mA.mC#mA#mG#**A**#mA#fC#fU#fC#fU

[1] m= 2’-O-methyl (2’-ribose modification), f= 2’-fluoro (2’-ribose modification), #= phosphorothioate (backbone modification), P= phosphate.
[2] Position 14 is shown in bold.
